# Comprehensive genome analysis of *Lentzea* reveals repertoire of polymer-degrading enzymes and bioactive compounds with clinical relevance

**DOI:** 10.1038/s41598-022-12427-7

**Published:** 2022-05-19

**Authors:** Pulak Kumar Maiti, Sukhendu Mandal

**Affiliations:** Laboratory of Molecular Bacteriology, Department of Microbiology, University of Calcutta, 35, Ballygunge Circular Road, Kolkata, 700019 India

**Keywords:** Computational biology and bioinformatics, Drug discovery, Microbiology

## Abstract

The genus *Lentzea* is a rare group of actinobacteria having potential for the exploration of bioactive compounds. Despite its proven ability to produce compounds with medical relevance, *Lentzea* genome analysis remains unexplored. Here we show a detailed understanding of the genetic features, biosynthetic gene clusters (BGCs), and genetic clusters for carbohydrate-active enzymes present in the *Lentzea* genome. Our analysis determines the genes for core proteins, non-ribosomal peptide synthetase condensation domain, and polyketide synthases-ketide synthase domain. The antiSMASH-based sequence analysis identifies 692 BGCs among which 8% are identical to the BGCs that produce geosmin, citrulassin, achromosin (lassopeptide), vancosamine, anabaenopeptin NZ857/nostamide A, alkylresorcinol, BE-54017, and bezastatin. The remaining BGCs code for advanced category antimicrobials like calcium-dependent, glycosylated, terpenoids, lipopeptides, thiopeptide, lanthipeptide, lassopeptide, lingual antimicrobial peptide and lantibiotics together with antiviral, antibacterial, antifungal, antiparasitic, anticancer agents. About 28% of the BGCs, that codes for bioactive secondary metabolites, are exclusive in *Lentzea* and could lead to new compound discoveries. We also find 7121 genes that code for carbohydrate-degrading enzymes which could essentially convert a wide range of polymeric carbohydrates. Genome mining of such genus is very much useful to give scientific leads for experimental validation in the discovery of new-generation bioactive molecules of biotechnological importance.

## Introduction

Microbes, specially actinobacteria, have been considered for a long time as the most valuable sources of secondary metabolites with pharmaceutical importance especially as antiviral, antibacterial, antifungal, antiparasitic agents, chemotherapeutics, immunomodulators, and cardiovascular drugs^1^. Isolation of such immensely important metabolites is mainly based on the extraction of culture filtrates, but this strategy was found to be insufficient as many compounds remain untouched and unexplored^2^. The genome sequence-based analyses of the depicted that many bacteria have cryptic biosynthetic gene clusters (BGCs) which failed to synthesize desired end products in laboratory conditions^3,4^. With the increasing threats of several potentially fatal diseases like cancer, alzheimer’s, cirrhosis, tuberculosis, pneumonia, influenza, etc., by rapidly emerging multi-drug resistant (MDR) pathogens, are also becoming more prevalent which makes it very necessary to find out new compounds with clinical relevance. In the last few years genome mining approaches are believed to be a new arsenal for the discovery of the clinically important biologically active molecules^5–7^. In-silico analysis of the bacterial draft or complete genome sequence data revealed the repertoire of several BGCs organized in close proximity in the genome. These adjacent genes code for enzymes involved in multiple metabolic pathways that are associated with various natural product biosynthesis^8^. Many chemical variants of the natural products originated from several BGCs, such as non-ribosomal peptides synthetases (NRPSs), polyketide synthases (PKSs), ribosomally synthesized and post-translationally modified peptides (RiPPs), saccharides, alkaloids and terpenoids. Analysis of these clusters helps to find out novel metabolites with a variety of chemical structures such as cationic peptides like brevicidine and laterocidine^9^; calcium-dependent antibiotics like cadasides^10^; lipopeptide antibiotics like taromycin A^11^; lantibiotics like roseocin^12^; glycopeptide antibiotics like mannopeptimycins^13^; thiopeptides like thiostrepton^14^; and lassopeptides like citrulassin^15^. Furthermore, this approach is emerged as useful in finding novel drugs as it seems to reduce time and cost of production, as well as diminish the redundancy of re-isolation of the same chemical compounds^4^.

The genus *Lentzea* is Gram-positive, aerobic, non-motile actinobacteria and have branched aerial mycelia which fragment into rod-shaped elements^16,17^. At the time of writing, it contains only 20 type species (one among them found to be two different subspecies) (https://lpsn.dsmz.de/search?word=lentzea) identified globally. The typical characteristics of this genus are the presence of meso-diaminopimelic acid in the cell wall, galactose, mannose and ribose as the predominant sugars in the whole cell lysate, straight-chain saturated or unsaturated and branched-chain saturated fatty acids in the cell, MK-9(H4) and phosphatidylethanolamine, diphosphatidylglycerol, phosphatidylglycerol and phosphatidylinositol as the major menaquinone and polar lipids, respectively, in the plasma membrane. The *Lentzea* contain 68.6–79.6% GC in their genome^16,17^. To our knowledge, the genome mining of this genus is not reported so far, therefore, this research could be an alternative choice for exploring new drug candidates. Moreover, bioactive compounds from this genus have not been reported much, with an exception of *Lentzea violacea* which found to be a promising source of antituberculosis compounds^18^. In our previous study, we have mentioned the taxonomic evaluation of novel species *Lentzea indica* PSKA42^T^^17^. Here we have focused on the genome sequence analysis of *L. indica* PSKA42 along with the genome of all available *Lentzea* for exploring the probable biosynthetic clusters, putative products by these BGCs, evolutionary relationship of PKS KS and NRPS C domains and CAZymes (carbohydrate-active enzymes) present therein. By screening 21 *Lentzea* genomes, we have identified a large number of gene clusters responsible for the biosynthesis of novel putative compounds of medicinal and agro-biological importance.

## Results

### General Properties of *Lentzea* Genome

Out of 21 genomes, only *L. guizhouensis* DHS C013 is a complete genome that has one contig (rest of the genomes contains > 30 contigs) and rest can be treated as draft genome. The genome length and contig number of all strains vary from 8.5 to 10.6 MB and 1 to 634, respectively. Among them, the smallest and largest genomes are *L. fradiae* CGMCC 4.3506 and *L. aerocolonigenes* NBRC 13195. The PGAP annotation revealed that the number of genes ranges from 8071 to 10,001, among them rRNAs, tRNAs and pseudogenes are ranges between 5 and 18, 61 and 71, 84 and 993 in number, respectively. All genomes are re-annotated by RAST where the number of features (size, GC%, number of coding sequences, number of RNAs) are different for different species (Table 1). The NCBI and RAST-based annotations have very nominal differences among themselves (Table 1, Table S1).

**Table 1:** Detailed characteristics of *Lentzea* genomes.

Organisms (accession no.)	*Size	*GC%	*N50	*L50	*Contig no (with PEGs)	Subsystems no	No. of Coding Sequences	No. of RNAs
*L. indica* PSKA42 (VSRL00000000)	99,67,719	68.3	30,279	94	634	328	10,535	68 (78)
*L. guizhouensis* DHS C013 (CP016793)	99,97,872	70	0	1	1	321	10,045	83 (91)
*L. albidocapillata* subsp *violacea* IMSNU 50388 (FNET00000000)	86,71,075	69	331,077	8	57	324	8495	72 (88)
*L. aerocolonigenes* NBRC 13195 (BBOJ01000000)	1,06,98,154	68.9	338,341^#a^	12	55	334	10,135	75 (80)
*L. albidocapilata* subsp. *albidocapillata* DSM 43393 (FWYC00000000)	86,39,486	68.7	397,314^#b^	9	37	321	8341	67 (79)
*L. californiensis* DSM 43393 (Go0103654)	89,98,498	69.3	528,830	6	34	329	8662	74 (87)
*L. flaviverrucosa* As40578 (QQAU00000000)	94,68,454	69.2	462,737	7	31	325	9192	73 (78)
*L. jiangxiensis* CGMCC 4.6609 (FNIX00000000)	85,91,279	70.2	279,756	7	61	320	8431	71 (83)
*L. xinjiangensis* CGMCC 4.3525 (FOFR00000000)	86,84,108	70.7	283,838	10	54	313	8530	68 (79)
*L. cavernae* CGMCC 4.7367 (BNAR00000000)	97,38,650	69.6	472,751	7	39	322	9464	68 (72)
*L. waywayandensis* DSM 44,232 (FOYL00000000)	1,01,53,412	68.9	475,994	6	33	335	9551	71 (86)
*L. atacamensis* DSM 45479 (QLTT00000000)	93,06,230	68.9	785,641	4	32	328	9292	76 (80)
*L. fradiae* CGMCC 4.3506 (FNCC00000000)	85,08,028	70.5	479,571	8	43	317	8236	65 (80)
*L. pudingi* CGMCC 4.7319 (BMNC00000000)	92,09,394	69.1	402,083^#c^	7	56	317	9076	71 (77)
*L. albida* DSM 44437 (FOFV00000000)	94,41,135	70.2	396,702^#d^	8	40	329	9017	68 (83)
*L. terrae* NEAU-LZS (QFWW00000000)	1,05,81,732	68.7	211,542	16	59	336	10,140	66 (76)
*L. kentuckyensis* NRRL B-24416 (MUYM00000000)	1,02,10,611	68.8	90,616^#e^	34	261	330	9648	68 (71)
*L. deserti* DSM 45480 (QGHB00000000)	95,29,573	68.8	442,485	7	41	322	9469	76 (79)
*L. flava* JCM 3296 (BMRE00000000)	97,21,257	69	133,734	20	188	333	9410	71 (81)
*L. nigeriaca* DSM 45680 (JAFBCX000000000)	93,32,327	68.9	641,913	6	42	334	8982	71 (79)
*L. alba* NEAU-D13 (JAAMPJ000000000)	1,02,11,123	68.7	431,430	8	35	329	9615	73 (82)

*Both from PGAP and RAST, numbers in parentheses of last column indicates results obtained from PGAP annotation data from NCBI.

^#^Difference in number between two annotation program.

^#a^348916.

^#b^397314.

^#c^432100.

^#d^352714.

^#e^92128.

### Comparative genomics among the *Lentzea* sp

**Figure 1:**
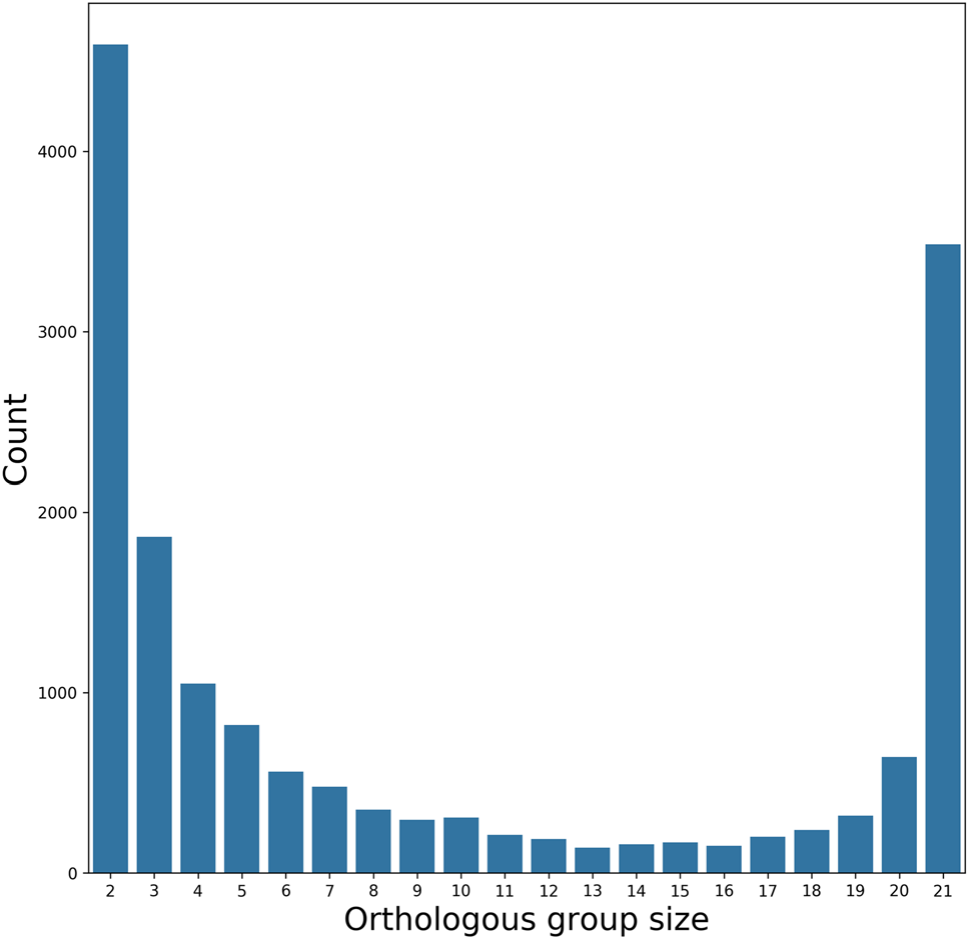
Distribution of orthologous genes present in *Lentzea* genomes.

The number of ORF present in *Lentzea* genomes ranges from 8037 to 9931, however, one strain possesses 10,311 ORFs (Figs. S1, S2). There are 3483 genes (i.e., core gene) shared among all *Lentzea* (Fig. 1).

According to the RAST database, the majority of genes present in all these genomes are involved in various functions which can be categorized mainly into 5 divisions such as (1) cofactors, vitamins, prosthetic groups, pigments; (2) protein metabolism; (3) fatty acids, lipids, and isoprenoids; (4) amino acids and derivatives; and (5) carbohydrate metabolism. The three most abundant genes are associated with biosynthesis of amino acids and derivatives, carbohydrate’s metabolism and cofactors, vitamins, prosthetic groups, and pigments, respectively. Interestingly we found that the two categories of genes, which codes for (1) cofactors, vitamins, prosthetic groups, pigments and (2) amino acids and derivatives, are highest in *L. indica* PSKA42 compared to other species. In addition, *L. indica* PSKA42 genome has second largest number of genes for nucleosides and nucleotides; phosphorus metabolism and third most for carbohydrate metabolism. All genomes contain minimum of 53 genes for mitigating oxidative, osmotic, metal-induced stress with the only exception of *L. albida* DSM 44437 genome which contains only 7 genes (Fig. S3). By this analysis none of the genomes were found to have photosynthetic genes or genes associated with cell division and cell cycle.

**Figure 2:**
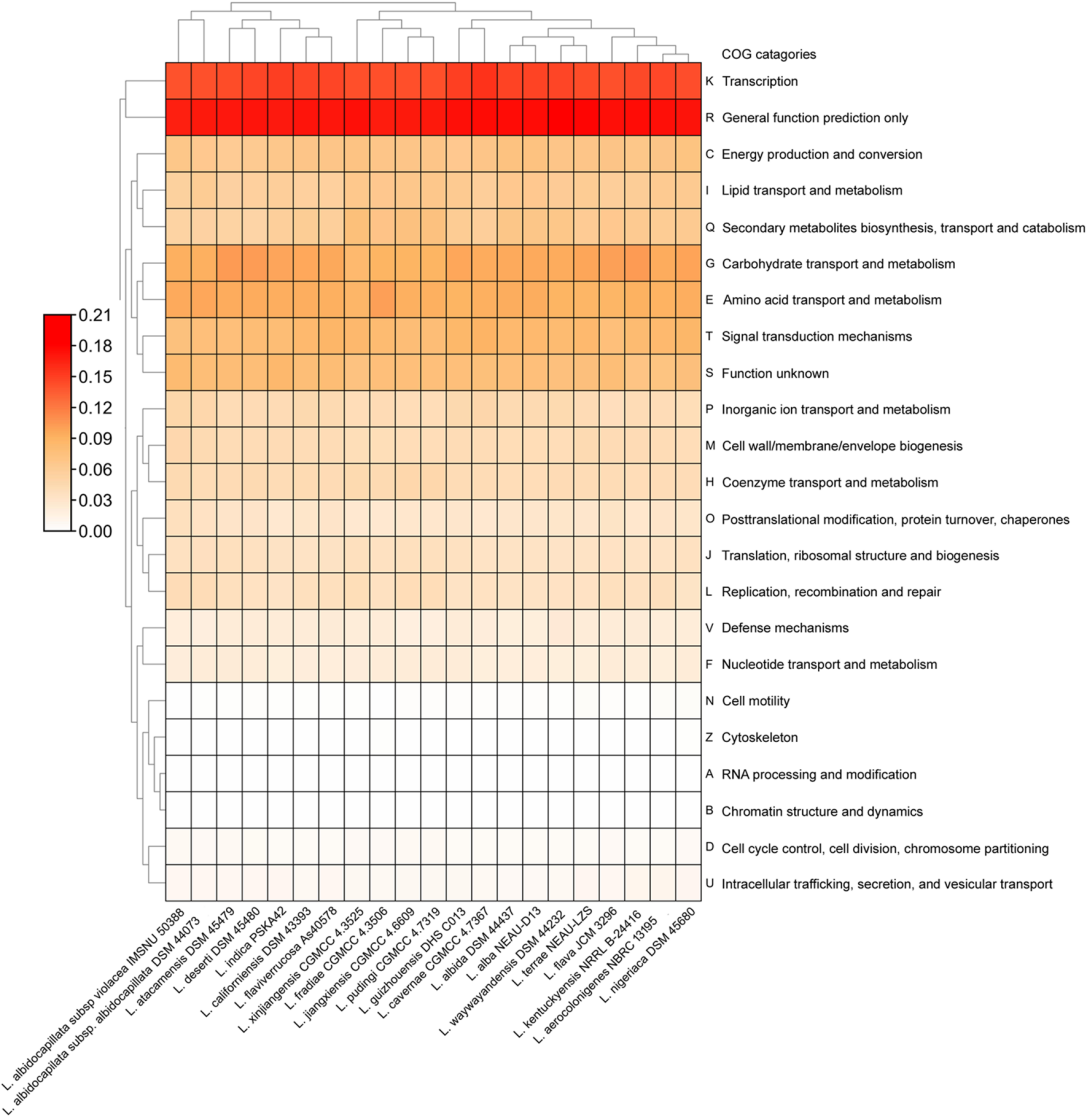
Functional classification of protein-coding genes present in *Lentzea* genomes by the abundance of Clusters of Orthologous Groups (COGs). The colour code represents the level of abundance.

Total 23 functional categories of COGs protein are observed which can be classified into four main groups including (1) information storage and processing [translation, ribosomal structure and biogenesis (J); RNA processing and modification (A); transcription (K); replication, recombination and repair (L); and chromatin structure and dynamics (B)]; (2) cellular processing and signalling [cell cycle control, cell division, chromosome partitioning (D); defence mechanisms (V); signal transduction mechanisms (T); cell wall/membrane/envelope biogenesis (M); cell motility (N); cytoskeleton (Z); intracellular trafficking, secretion, and vesicular transport (U); and posttranslational modification, protein turnover, chaperones (O)]; (3) metabolism [energy production and conversion (C); carbohydrate transport and metabolism (G); amino acid transport and metabolism (E); nucleotide transport and metabolism (F); coenzyme transport and metabolism (H); lipid transport and metabolism (I); inorganic ion transport and metabolism (P); and secondary metabolites biosynthesis, transport and catabolism (Q)]; and (4) poorly characterized [general function prediction only (R) and function unknown (S)] in the *Lentzea* genomes (Fig. 2). Among the 23 COG proteins, the most abundant protein is engaged in general cellular function (R) and belongs to the poorly characterized group with an average abundance of 0.17. The proteins for information storage and processing show abundances ranging between 0.0001 and 0.1566. The transcription-related proteins (K) under the information storage and processing group of proteins, show an average abundance of 0.145 with exception of having even above 0.15 average abundance in *L. indica* PSKA42, *L. guizhouensis* DHS C013, and *L. cavernae* CGMCC 4.7367. Out of the cellular processes and signaling proteins, signal transduction mechanisms protein (T) has the highest abundance with an average of 0.0824. The COGs categories related to metabolism responsive proteins are accounted for ranges between 0.01 and 0.1, among them carbohydrate and amino acid transport and metabolism-related proteins (G, E) are most abundant with an average of 0.09. Four species including *L. atacamensis* DSM 45479, *L. kentuckyensis* NRRL B-24416, *L. deserti* DSM 45480, and *L. flava* JCM 3296 show a higher abundance (0.1) for carbohydrate transport and metabolism-related proteins (G).

All genomes contain 34–38% annotated genes, which are functionally divided into six categories: metabolism, genetic information processing, environmental information processing, cellular processes, organismal systems, and related to human diseases according to the KEGG database. This KEGG pathway analysis showed that a number of genes of the above categories differ in each species (Fig. S4). Among them, majority are engaged in metabolism and rests are assigned to environmental information processing. These six categories are divided into many divisions and subdivisions. For example, the ‘metabolism’ category contains a subdivision known as ‘global and overview maps’ which contributed around 45% (1730 genes on average) of all these six categories. Carbohydrate and amino acid metabolism groups (under the metabolism category) are responsible for roughly 8.5% (average 330 genes) and 7.8% (average 300 genes) of the total genes, respectively. Also, four different functional classes such as energy metabolism; metabolism of cofactors and vitamins; xenobiotics biodegradation and metabolism (under metabolism) and membrane transport (under environmental information processing) are contributed by 100–159 genes from the total. The *L. terrae* NEAU-LZS contains the highest number of genes for metabolism, environmental information processing and cellular activities processes.

**Figure 3:**
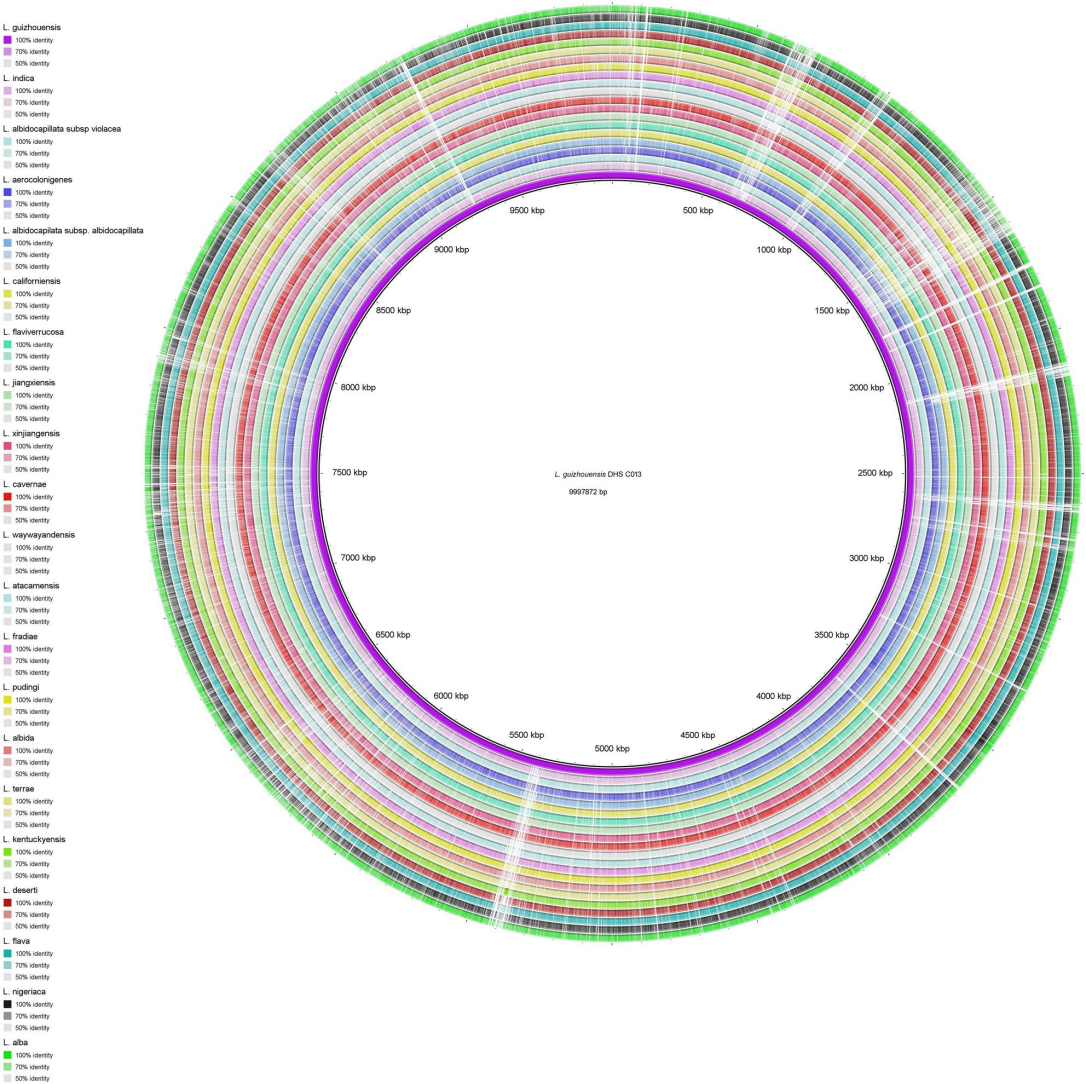
Circular map of the complete genome of *L. guizhouensis* DHS C013 along with the comparative genome maps of rest of the available *Lentzea* genome sequences. The figure was designed using BRIG^25^. The gaps in the circles represent regions of low or no similarity.

**Figure 4:**
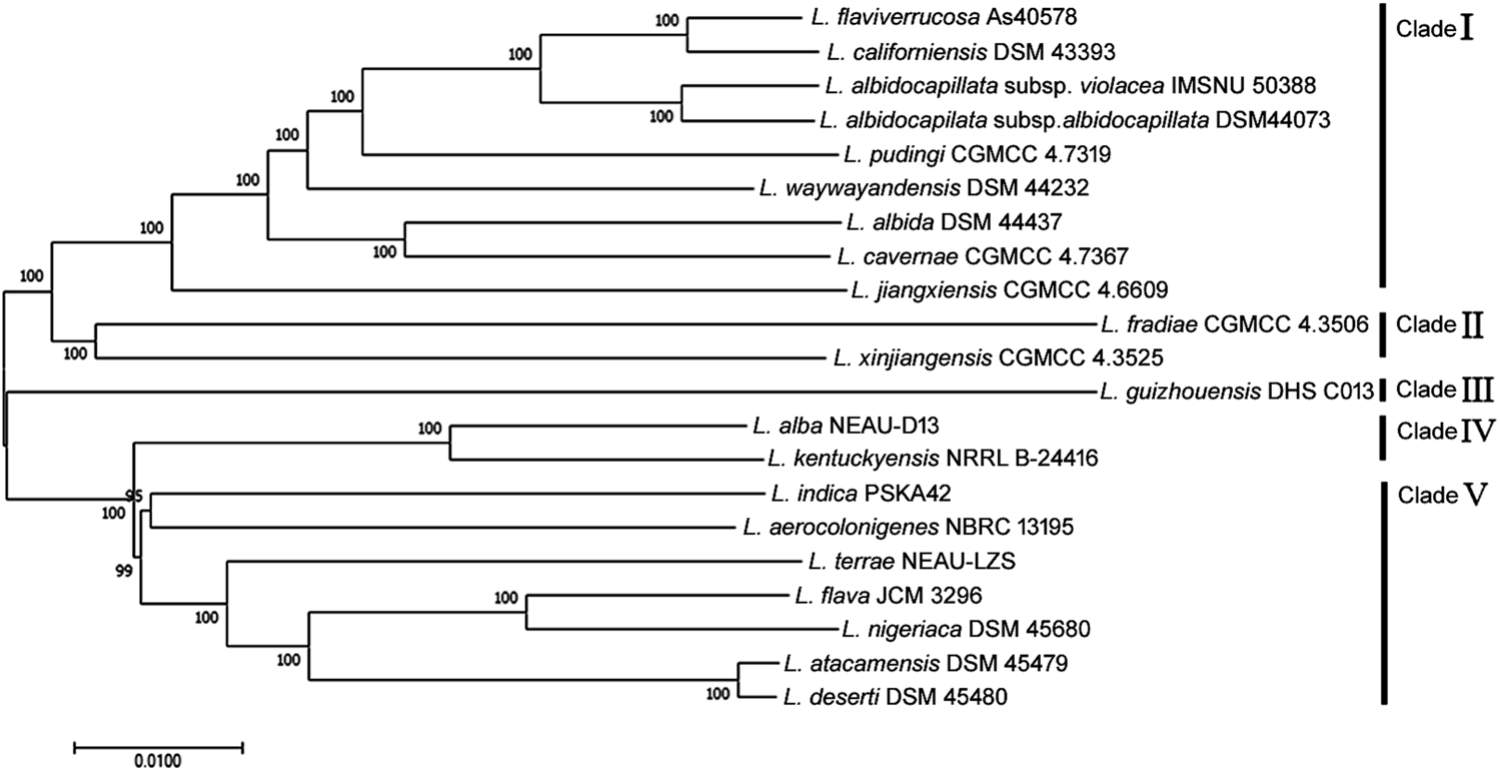
Neighbor-joining phylogenetic relationship based on core proteome of *Lentzea* species. Numbers at nodes refer to bootstrap values based on 1000 replicates. Bar, 0.01 means amino acid substitutions per 100 amino acid position.

The *L. guizhouensis* DHS C013 is the only complete genome and was used to compare against other genomes through a circular map generated by BLAST + method. The map showed gaps or low similarity regions that indicate variations in numerous regions (Fig. 3). All species and subspecies are well separated from each other based on ANI, dDDH values, except *L. atacamensis* DSM 45479, *L. deserti* DSM 45480. The percentage of ANIm, ANIb and dDDH among these two species is 98.82, 98.23 and 88.6, respectively, which is above than recommended cut off (94–96%, 94–96%, and 70%, respectively for species level and 98%, 98%, and 79%, respectively for subspecies level)^19–22^. Thus, it was found justified to merge these two species under a single species. At the time of manuscript preparation, these two species were claimed as a single species by Ping et al. (2021)^23^. Phylogenetic analysis based on the core proteome data exhibited that all strains can be formed in five clades (I-V). Clade I, II, III, IV, V contains 9 (*L. flaviverrucosa* As40578, *L. californiensis* DSM 43393, *L. albidocapillata* subsp *violacea* IMSNU 50388, *L. albidocapilata* subsp. *albidocapillata* DSM 43393, *L. pudingi* CGMCC 4.7319, *L. waywayandensis* DSM 44232, *L. albida* DSM 44437 *L. cavernae* CGMCC 4.7367 *L. jiangxiensis* CGMCC 4.6609); 2 (*L. fradiae* CGMCC 4.3506, *L. xinjiangensis* CGMCC 4.3525); 1 (*L. guizhouensis* DHS C013); 2 (*L. alba* NEAU-D13, *L. kentuckyensis* NRRL B-24416); 7 (*L. indica* PSKA42, *L. aerocolonigenes* NBRC 13195, *L. terrae* NEAU-LZS, *L. flava* JCM 3296 *L. nigeriaca* DSM 45680, *L. deserti* DSM 45480, *L. atacamensis* DSM 45479), respectively (Fig. 4). Phylogenomic analysis performed by TYGS also recovered most of the clades as core protein-based phylogenetic analysis (Fig. S5).

### Biosynthetic gene clusters of *Lentzea* species

**Figure 5:**
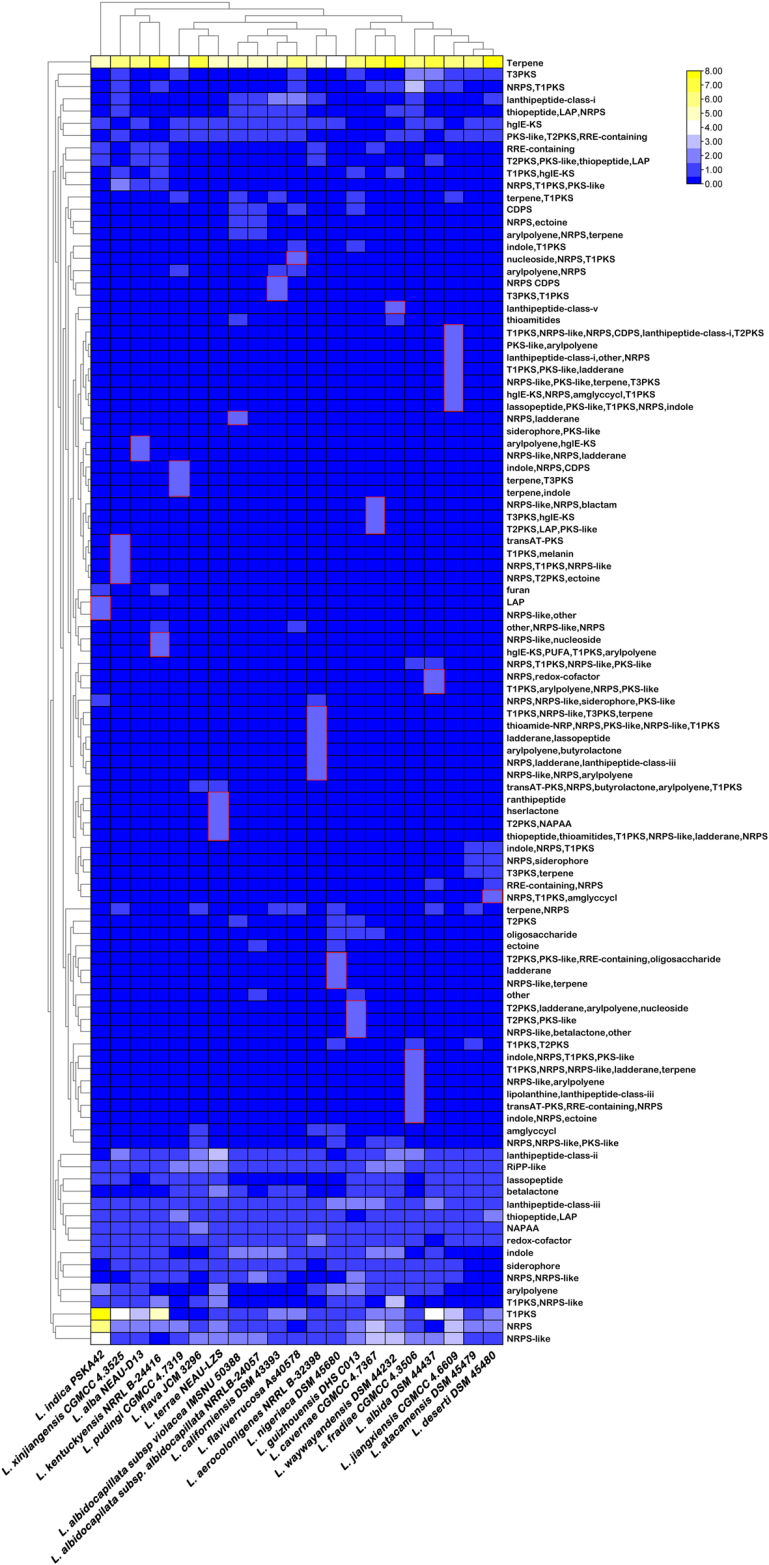
Heat map showing the abundance of BGCs distributed in *Lentzea* genome as predicted by antiSMASH database. Color keys represent variation of copy numbers of individual cluster among *Lentzea* genome.

**Figure 6:**
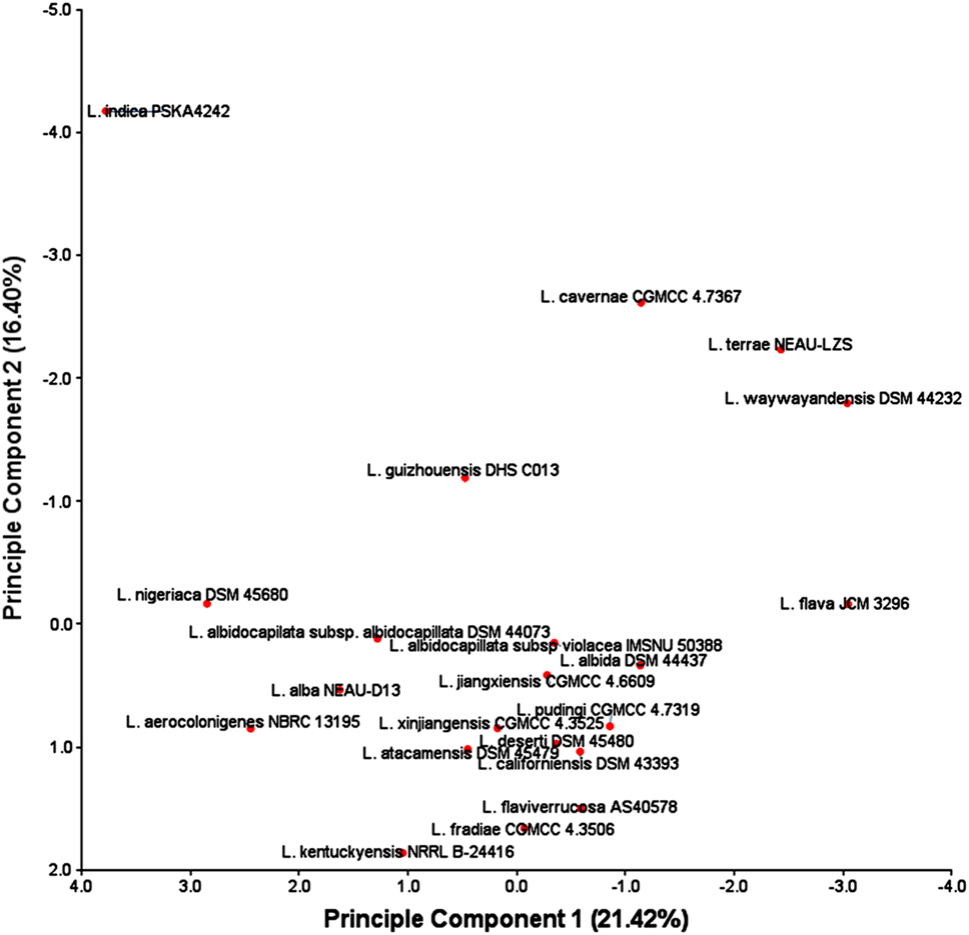
Principal component analysis (PCA) among *Lentzea* species based on BGCs recovered through antiSMASH for their relationship.

It has been found that a total of 692 superclusters (region) are analyzed by antiSMASH, among which the highest number (40) is present in *L. indica* PSKA42 and *L. waywayandensis* DSM 44232, while the lowest number of BGCs is 26 in *L. atacamensis* DSM 45479. The unique species-specific cluster present in all 18 different species (except *L. albidocapilata* subsp. *albidocapillata* DSM 44073, *L. atacamensis* DSM 45479, and *L. flava* JCM 3296) (Fig. 5, Fig. S6). This study conveys that species of *Lentzea* are the repertoires of valuable components of pharmaceutical significance because 692 cluster represents various secondary metabolites producing genes including polyketide synthases type I (PKSI) (45), non-ribosomal peptide synthetase (NRPS) (37), NRPS-like (36), hybrid cluster of polyketides (PKS/NRPS) with others (185), RiPP (thiopeptide, lanthipeptide, lassopeptide, ranthipeptide, thioamitides, LAP) (77), RiPP-like (26). Out of 185 hybrid clusters, 37 clusters are from RiPP hybrid cluster. Also, clusters for common metabolites are found in all species which includes clusters for terpene (124), redox-cofactor (21), and NAPAA (22). Out of 692 clusters, other important BGCs are for siderophore (17), arylpolyene (15), indole (20), hglE-KS (15), betalactone (14), T3PKS (11), RRE-containing (5), CDPS (4), T2PKS (3), oligosaccharide (3), amglyccycl (3), furan (2), ectoine (2), ladderane (1), hserlactone (1), transAT-PKS (1), and other (2) (Fig. S6, Dataset S1). This result articulates that although the genomes of various *Lentzea* species harbour multiple common clusters but the number could vary from species to species (Fig. 5). Similar to cladogram derived on BGCs data, also PCA clearly conveyed the consistent relationship among the *Lentzea* as strain PSKA42 is found in both cases in distinct positions from others (Fig. 6).

### The highly similar BGCs codes for the putative products

Out of 692, 502 BGCs are related to the known diverse compounds. All of the highly similar compounds were chemically characterized and were present in the Minimum Information about a Biosynthetic Gene cluster (MiBIG) database which was determined directly through antiSMASH. We have divided all identified cluster encoded important putative products into two sections viz*.* highly similar (50–100%) and less similar (< 50%).

### Antimicrobial

**Figure 7:**
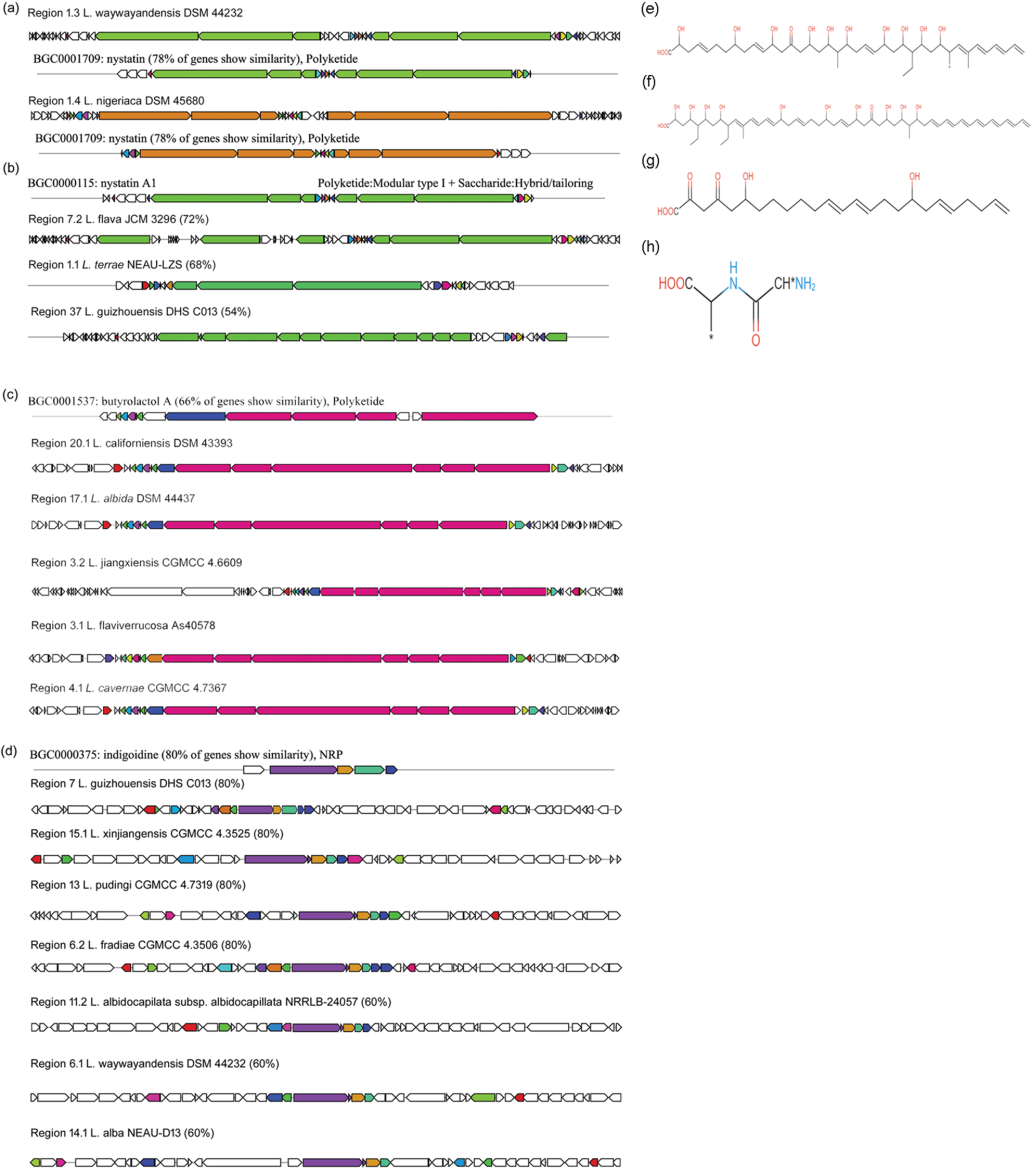
Highly similar antimicrobial gene clusters of *Lentzea* species compared with known clusters in the antiSMASH database. Gene clusters for Nystatin (**a**), Nystatin A1 (**b**), Butyrolactol A (**c**), Indigoidine (**d**); and the putative compounds produced by these clusters Nystatin (**e**), Nystatin A1 (**f**), Butyrolactol A (**g**), and Indigoidine (**h**).

Genomes of *L. nigeriaca* DSM 45680 and *L. waywayandensis* DSM 44232 contain a hybrid cluster that shows 78% similarity (Fig. 7a) with the cluster that codes for nystatin in *Streptomyces albulus* (T1 and NRPS like)^24^. The hybrid clusters of the above two genomes consist of > 1,70,000 nt comprising of 50 to 52 ORFs among which six are for core biosynthetic genes. These six core genes and other regulatory and accessory genes have been found similar to the genes that are involved in the biosynthesis of nystatin. The structure of these core genes (1–6) is made up of six modules with domain KS-AT-DH-KR-ACP, KS-AT-KR-ACP, KS-AT-KR-ACP, KS-AT-KR-ACP, KS-AT-KR-ACP, and KS-AT-KR-ACP-PKS_Docking_C term; four modules with domain KS-AT-DH-KR-ACP, KS-AT-KR-ACP, KS-AT-DH-ER-KR-ACP, and KS-AT-DH-KR-ACP-PKS_Docking_C term; one module with KS-AT-KR-PP-TE; two modules with domain CAL-KR-ACP, and KS-AT-KR-PP-PKS_Docking_C term; six modules with domain KS-AT-DH-KR-ACP, KS-AT-DH-KR-ACP, KS-AT-DH-KR-ACP, KS-AT-KR-PP, KS-AT-KR-ACP, and KS-AT-KR-ACP-PKS_Docking_C term; six modules with KS-AT-DH-KR-ACP, KS-AT-DH-KR-ACP, KS-AT-DH-KR-ACP, KS-AT-DH-KR-ACP, KS-AT-DH-KR-ACP, KS-AT-DH-KR-ACP-PKS_Docking_C term) for 1, 2, 3, 4, 5 and 6 core, respectively. Polymer prediction by aforementioned cluster is—(ccmal – ccmal – ccmal – ccmal – ccmal – ccmal) + (ccmal – ohmal – Me-ohmal – ohmal – mal – ohmal) + (ccmal – ohmal – redmal – ccmal) + (ohmal) + (ccmal – ccmal – Me-ccmal – ohemal – ohmal – ohemal) + (ohmal) and putative structure is given Fig. 7e. The standard nystatin biosynthetic gene cluster contains six core genes *nysI, nysJ, nysK, nysA*, *nysB* and *nysC* but the *L. nigeriaca* DSM 45680 and *L. waywayandensis* DSM 44232 genome have different domains/modules except for *nysI*. These *nysI*, *nysJ*, *nysK*, *nysA, nysB* and *nysC* genes match respectively with the core genes 1, 2, 3, 4, 5 and 6 present in above two genomes. Similarly, another derivative of nystatin known as nystatin A1 is found in *L. guizhouensis* DHS C013, *L. flava* JCM 3296, *L. terrae* NEAU-LZS) (Fig. 7b,f). Other T1PKS cluster encoded putative antifungal butyrolactol A has been identified (Fig. 7c,g) in five species such as *L. flaviverrucosa* As40578, *L. californiensis* DSM 43393, *L. albida* DSM 44437*, L. jiangxiensis* CGMCC 4.6609*, L. cavernae* CGMCC 4.7367. The related genomes differ in modules because these genes show only 66% identity. The antimicrobial compound indigoidine produced from NRPS like clusters of *Streptomyces chromofuscus*^25^ and shows 80% similarity with that of the *L. guizhouensis* DHS C013, *L. fradiae* CGMCC 4.3506, *L. pudingi* CGMCC 4.7319, *L. xinjiangensis* CGMCC 4.3525 and 60% with *L. albidocapilata* subsp. *albidocapillata* DSM 44073, *L. waywayandensis* DSM 44232 and *L. alba* NEAU-D13 (Fig. 7d,h). The domain organization of this compound is same as the chemically characterized compound except the one from *L. guizhouensis* DHS C013 which contains an extra TE domain. This compound is a natural by-product and can be used as an alternative blue dye with antioxidant activity.

### Chemotherapeutic/anticancer compounds

**Figure 8:**
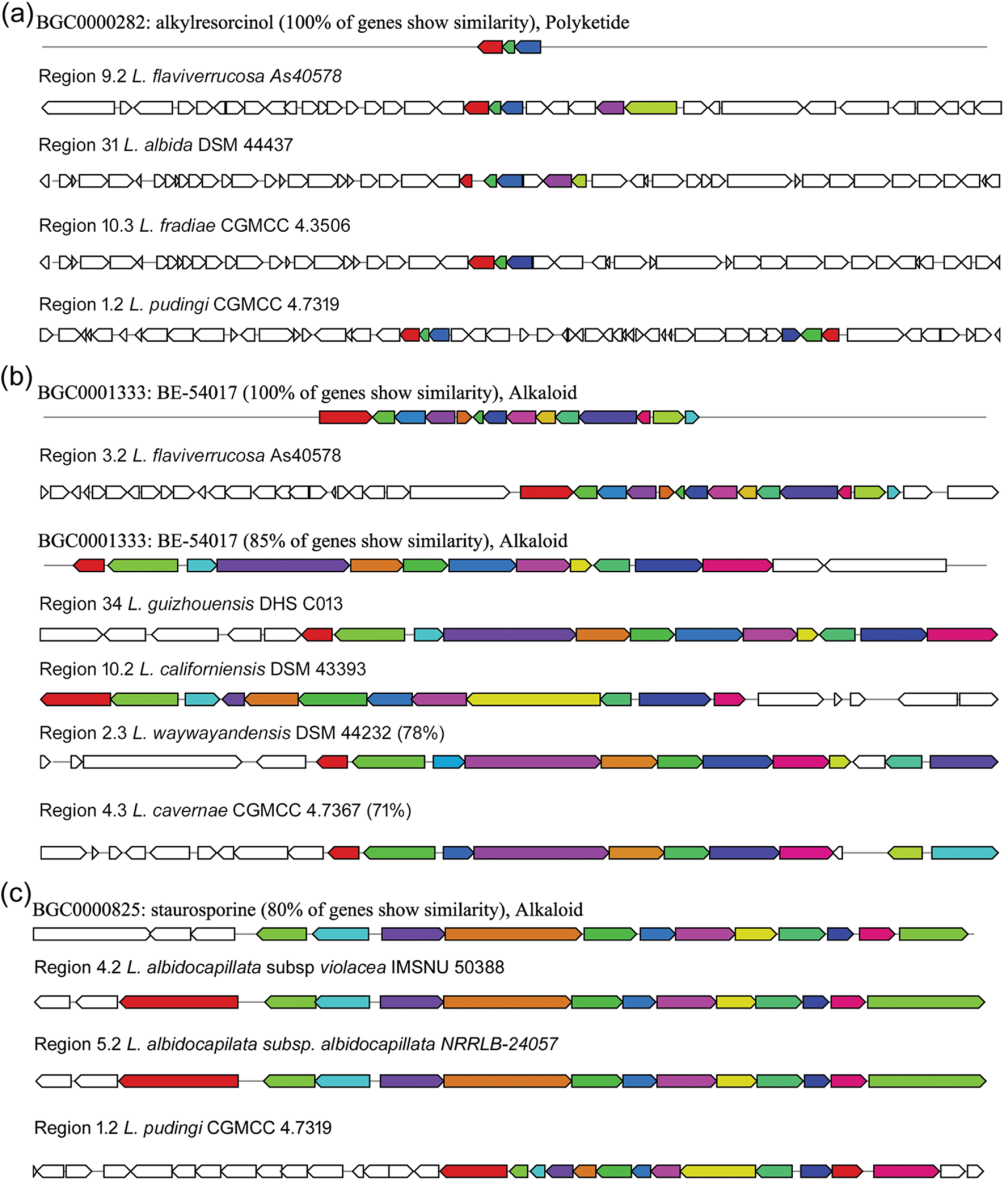
Highly similar antitumor gene clusters of *Lentzea* species compared with known clusters in the antiSMASH database. (**a**) alkylresorcinol, (**b**) BE-54017, (**c**) staurosporine.

There are alkylresorcinol coding T3PKS clusters present in the genome of four *Lentzea* species (Fig. 8a) having 100% sequence similarity with *Streptomyces griseus* subsp. *griseus* NBRC 13350^26^. This product naturally presents in many cereals that can show invitro anticancer properties. Hybrid polyketide cluster (T1PKS, indole) that codes BE-54017 (Fig. 8b), have 71–100% similarity with the uncultured bacterium AB1650. Both domains are same and this natural product is a small family of indolotryptoline which shows activity against tumour cell lines^27^. Another compound staurosporine (Alkaloid) is found in three *Lentzea* genomes and has 80% sequence similarity to the known clusters (Fig. 8c).

### Siderophores

**Figure 9:**
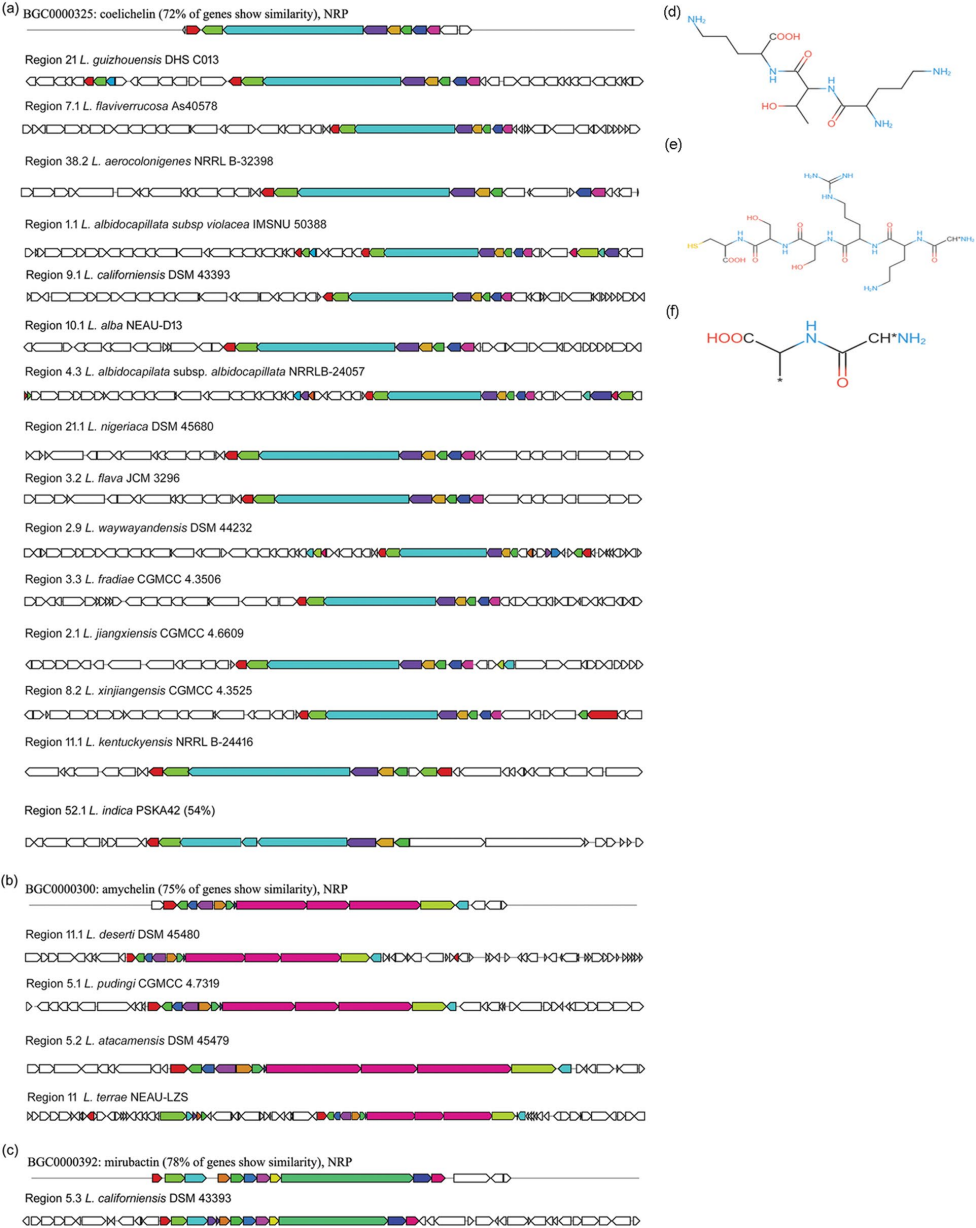
Highly similar siderophores gene clusters of *Lentzea* species compared with known clusters in the antiSMASH database. Gene clusters for coelichelin (**a**), amychelin (**b**), mirubactin (**c**); and the putative compounds produced by these clusters coelichelin (**d**), amychelin (**e**), and mirubactin (**f**).

Out of 21 *Lentzea* species, 15 harbour of coelichelin type siderophore compound (Fig. 9a,d) which was originally isolated from another actinobacterium, *Streptomyces coelicolor.* The NRPS clusters of these genomes show 72% identity (except *L. indica* PSKA42 (54%) with that of the *S. coelicolor* and the predicted polymer by these genomes is D-orn—D-thr—orn. This peptide siderophore compound (domain A-PCP-E, C-A-PCP-E, and C-A-PCP) is the same among species that harbour it, but the low identity indicates that some of these might code for a novel compound. Few genomes also contain two different types of siderophores such as amychelin (NRPS), and mirubactin (NRPS) which are more than 75% similar to the known compounds (Fig. 9b,c,e,f). Known amychelin biosynthetic gene clusters of *Streptomyces* sp. AA4 and query genome are comprised of the same domains like PP-C-A-PCP, C-A-PCP, C-A-PCP-E, C-A-PCP-E, C-A-PCP, C-A-PCP-E-NRPS_COM_Cterm, Adenylation domain). The rough predicted polymer codes by this putative cluster are (X − D-orn) + (D-arg) + (ser − D-ser) + (cys). The product mirubactin domain of *Actinosynnema mirum* DSM 43,827 (C-A-PCP-E, C-A-PCP-E, E, A, KR) is also similar to *L. californiensis* only except the E domain.

### Lower similarity (> 50%) BGCs encoded products

#### Chemotherapeutic/anticancer compounds

All genomes contain genes for two putative compounds named LL-D49194α1 (LLD) and lankacidin C encoded by the mixture of polyketide genes and are the probable antitumor agents. These particular loci of all *Lentzea* genomes show 30–50% similarity (except for two strains *L. pudingi* CGMCC 4.7319 and *L. guizhouensis* DHS C013 showing only 3% similarity) with *Streptomyces vinaceusdrappus* LLD biosynthetic gene cluster. Nine genomes (*L. guizhouensis* DHS C013*, L. flaviverrucosa* As40578, *L. californiensis* DSM 43393, *L. albidocapilata* subsp. *albidocapillata* NRRLB-2405, *L. cavernae* CGMCC 4.7367, *L. pudingi* CGMCC 4.7319, *L. flava* JCM 3296, *L. jiangxiensis* CGMCC 4.6609, *L. xinjiangensis* CGMCC 4.3525) harbour genes that code for putative tetracycline polyketide product SF2575 but shows only 4–6% similarity with the standard terpenoid polyketide product cluster. Another T1PKS encodes antitumor agent tiancimycin and is found to be present in genomes (16–22% similar) of *L. albidocapilata* subsp. *violacea* IMSNU 50388*, L. albidocapilata* subsp. *albidocapillata* DSM 44073*, L. atacamensis* DSM 45479*, L. deserti* DSM 45,480*,* and *L. waywayandensis* DSM 44232. The bleomycin producing genes are also present in *L. albidocapilata* subsp. *albidocapillata* DSM 44073*, L. waywayandensis* DSM 44232*, L. kentuckyensis* NRRL B-24416 but the similarity is only 6–12%. Tallysomycin coding gene has 5% similarity with the one present in the genome of *L. waywayandensis* DSM 44232, *L. californiensis* DSM 43393. Cheamicin producing genes (6% similar, T1PKS) are present in *L. guizhouensis* DHS C013 and *L. kentuckyensis* NRRL B-24416. Cetoniacytone A is similar (9%) with *L. flava* JCM 3296, *L aerocolonigens* and *L. nigeriaca* DSM 45680. Three genomes contain genes (*L. jiangxiensis* CGMCC 4.6609, *L. cavernae* CGMCC 4.7367, *L. californiensis* DSM 43393) that are found to be similar (28%) with the polyketide encoded (T1PKS) product maduropeptin. Furthermore, T1PKS, NRPS, NRPS-like gene clusters and other cluster codes for antitumor agents such as haliamide, elaiophylin (*L. guizhouensis* DHS C013), dynemicin A (*L. flaviverrucosa* As40578), actinomycin D (*L. jiangxiensis* CGMCC 4.6609), BD-12 (*L. cavernae* CGMCC 4.7367, *L. deserti* DSM 45480), herbimycin A (*L. terrae* NEAU-LZS); JBIR-126 (*L. indica* PSKA42) and tubulysin, herboxidiene (*L. waywayandensis* DSM 44232), lactonamycin Z (*L. nigeriaca* DSM 45680, *L. terrae* NEAU-LZS), CC-1065 (*L. albidocapilata* subsp. *albidocapillata* DSM 44073) has been detected (Dataset S1).

#### Antibacterial

The putative terpene gene cluster shares only 5–6% similarity with gene clusters expressing platencin in 11 different species such as *L. guizhouensis* DHS C013, *L. californiensis* DSM 43393, *L. flaviverrucosa* As40578, *L. albidocapilata* subsp. *albidocapillata* DSM 44073, *L. albidocapilata* subsp *violacea* IMSNU 50388, *L. atacamensis* DSM 45479, *L. deserti* DSM 45480, *L. waywayandensis* DSM 44232, *L. pudingi* CGMCC 4.7319, *L. jiangxiensis* CGMCC 4.6609, *L. albida* DSM 44437, and *L. terrae* NEAU-LZS. Fortimicin biosynthesizing cluster has been observed in 17 *Lentzea* species (other than *L. flava* JCM 3296, *L. pudingi* CGMCC 4.7319, *L. cavernae* CGMCC 4.7367, *L. fradiae* CGMCC 4.3506). The calcium dependent antibiotics such as glycinocin A (present in *L. albidocapilata* subsp. *albidocapillata* DSM 44,073, *L. cavernae* CGMCC 4.7367), CDA1b/CDA2a/CDA2b/CDA3a/CDA3b/CDA4a/CDA4b (present in *L. atacamensis* DSM 45479), cadaside A/cadaside B (present in *L. fradiae* CGMCC 4.3506, *L. kentuckyensis* NRRL B-24416, *L. pudingi* CGMCC 4.7319) and lipopeptide antibiotic A54145 ( present in *L. cavernae* CGMCC 4.7367), taromycin A (present in *L. waywayandensis* DSM 44232) rishirilide B/rishirilide A (present in *L. albida* DSM 44437), friulimicin A/friulimicin B/friulimicin C/friulimicin D (present in *L. albida* DSM 44437, *L. albidocapilata* subsp. *albidocapillata* DSM 44073) from different cluster has been detected. The cyclic peptide including RP-1776 (present in *L. jiangxiensis* CGMCC 4.6609), lydicamycin (present in *L. xinjiangensis* CGMCC 4.3525), dechlorocuracomycin (present in *L. xinjiangensis* CGMCC 4.3525, *L. pudingi* CGMCC 4.7319), telomycin (present in *L. guizhouensis* DHS C013), xantholipin (present in *L. nigeriaca* DSM 45680) and glycopeptide such as mannopeptimycin (present in *L. atacamensis* DSM 45479, *L. pudingi* CGMCC 4.7319, *L. waywayandensis* DSM 44232), kistamicin A (present in *L. indica* PSKA42), avoparcin (present in *L. albidocapilata* subsp. *albidocapillata* DSM 44073). The compound formicamycins A-M (present in *L. guizhouensis* DHS C013, *L. fradiae* CGMCC 4.3506), ulleungmycin (present in *L. indica* PSKA42, *L. guizhouensis* DHS C013, *L. californiensis* DSM 43393), enduracidin (present in *L. guizhouensis* DHS C013, *L. aerocolonigenes* NBRC 13195, *L. fradiae* CGMCC 4.3506, *L. jiangxiensis* CGMCC 4.6609), salinomycin (present in *L. indica* PSKA42, *L. guizhouensis* DHS C013, *L. kentuckyensis* NRRL B-24416, *L. alba* NEAU-D13), stenothricin (present in *L. flava* JCM 3296, *L. xinjiangensis* CGMCC 4.3525) maklamicin, (present in *L. aerocolonigenes* NBRC 13195, *L. xinjiangensis* CGMCC 4.3525), and virginiamycin S1 (present in *L. jiangxiensis* CGMCC 4.6609) from different clusters has also been found. Some species-specific clusters encode antibiotics with lower similarity levels such as azicemicin B, hygrocin A/hygrocin B, (present in *L. guizhouensis* DHS C013); thiolutin (present in *L. californiensis* DSM 43393); kosinostatin (present in *L. albidocapilata* subsp *violacea* IMSNU 50388); rifamorpholine A/rifamorpholine B/rifamorpholine C/rifamorpholine D/rifamorpholine E (present in *L. nigeriaca* DSM 45680); pseudouridimycin (present in *L. atacamensis* DSM 45479); limazepine C/limazepine D/limazepine E/limazepine F/limazepine A, (present in *L. cavernae* CGMCC 4.7367); aldgamycin J/aldgamycin K/aldgamycin P/aldgamycin E, mannopeptimycin (present in *L. deserti* DSM 45480), diazepinomicin (present in *L. albida* DSM 44437), terpenoid antibiotic brasilicardin A (present in *L. fradiae* CGMCC 4.3506); methylenomycin A, abyssomicin C/atrop-abyssomicin C, sipanmycin (present in *L. kentuckyensis* NRRL B-24416) has also been found (Dataset S1).

#### Antimycobacterial

Three antimycobacterial agents have been detected with a low level of identity (14–34%) such as viomycin (present in *L. indica* PSKA42), atratumycin (present in *L. guizhouensis* DHS C013, *L. albidocapilata* subsp. *violacea* IMSNU 50388, *L. aerocolonigenes* NBRC 13195), capreomycin IA/capreomycin IB/capreomycin IIA/capreomycin IIB (present in *L. cavernae* CGMCC 4.7367, *L. terrae* NEAU-LZS, *L. pudingi* CGMCC 4.7319) (Dataset S1).

#### Antifungal

Chemically characterized rustmicin producing gene cluster is only 10% similar to that of the *L. nigeriaca* DSM 45680, *L. atacamensis* DSM 45479, *L. deserti* DSM 45480, *L. fradiae* CGMCC 4.3506. The compound ECO-02301 encoding gene cluster has overall sequence similarity that ranges from 25 to 32% with the genome of *L. waywayandensis* DSM 44232, *L. xinjiangensis* CGMCC 4.3525, and *L. terrae* NEAU-LZS. Regions of *L. albida* DSM 44437, *L. kentuckyensis* are similar (5–7%) to the cluster of Sch-47554/Sch-47555. The genomes of *L. indica* PSKA42 and *L. waywayandensis* DSM 44232 contain PKS T1 gene clusters which are similar to the known cluster of caniferolide A/caniferolide B/caniferolide C/caniferolide D. The genes for compounds bacillomycin D, caerulomycin A, yatakemycin and nystatin/nystatin A1 exhibit similarity with *L. guizhouensis* DHS C013, *L. aerocolonigenes* NBRC 13195, *L. xinjiangensis* CGMCC 4.3525 and *L. albida* DSM 44437. Strain *L. jiangxiensis* is the reservoirs of several compounds like ibomycin, naphthomycin A (having antibacterial, antifungal, and antitumor activities), jawsamycin, cyphomycin, etc. Similarly, *L. fradiae* CGMCC 4.3506 contains phthoxazolin, cyphomycin, atratumycin. The nystatin-like *Pseudonocardia* polyene has been found to present in *L. indica* PSKA42, *L. fradiae* CGMCC 4.3506, *L. albida* DSM 44437 (Dataset S1).

#### Antiviral

The antiviral compound pyrazomycin (used as an anticancer agent) coding gene clusters show 8% sequence similarity to regions of *L. fradiae* CGMCC 4.3506 and *L. terrae* NEAU-LZS. Another antifungal, antitumor and antiviral hybrid polyketide cluster encoded compound 9-methylstreptimidone show 18–25% sequence similarity with *L. terrae* NEAU-LZS and *L. nigeriaca* DSM 45680. Some unique genes related to the antiviral compounds such as keratinimicin (also antibacterial properties, T1PKS, terpene), valinomycin/montanastatin (arylpolyene type), quartromicin A1, (hybrid of PKS and NRPS like) xiamycin A (terpene type), echinomycin (also has antibacterial, anticancer, NRPS like activity) found exclusively in specific strains like *L. albidocapilata* subsp. *violacea* IMSNU 50388, *L. cavernae* CGMCC 4.7367, *L. waywayandensis* DSM 44232, *L. xinjiangensis* CGMCC 4.3525, *L. flava* JCM 3296, respectively (Dataset S1).

#### Insecticidal/antiparasitic

Few gene clusters code for insecticidal such as meilingmycin, aculeximycin, and paromomycin are strain-specific and found with < 26% sequence similarity in *L. waywayandensis* DSM 44232, *L. albida* DSM 44437, *L. xinjiangensis* CGMCC 4.3525, respectively. The antiparasitic compound clusters of sipanmycin, lobosamide A/lobosamide B/lobosamide C (against *Trypanosoma brucei*), catenulisporolides (against *Plasmodium falciparum*) have < 20% similarity with *L. kentuckyensis, L. xinjiangensis* CGMCC 4.3525, *L. fradiae* CGMCC 4.3506, and *L. xinjiangensis* CGMCC 4.3525, respectively (Dataset S1).

#### Siderophore

Two clusters responsible for the production of siderophore compounds such as qinichelins (by a hybrid cluster of NRPS), coelibactin (NRPS and T1) are found in *L. nigeriaca* DSM 45680; *L. flaviverru*, and *L. fradiae* CGMCC 4.3506. As mentioned earlier, 17 more siderophore clusters are present in 17 *Lentzea* species (except *L. aerocolonigenes* NBRC 13195, *L. indica* PSKA42, *L. atacamensis* DSM 45479, *L. deserti* DSM 45480) but have no known product except yatakemycin in *L. xinjiangensis* CGMCC 4.3525 (Dataset S1).

### Other biological active/natural product

The T1 PKS and hglE-KS clusters are found which are similar to the clusters responsible for industrially important compounds like apoptolidin (also present in the genome of *L. indica* PSKA42), vazabitide A (also present in the genome of *L. californiensis* DSM 43393), rishirilide B/rishirilide A (also present in the genome of *L. nigeriaca* DSM 45680); divergolide A/divergolide B/divergolide C/divergolide D (also present in the genome of *L. pudingi* CGMCC 4.7319 and *L. californiensis* DSM 43393), microansamycin, xiamycin A (also present in the genome of *L. albida* DSM 44437), akaeolide (also present in the genome of *L. aerocolonigenes* NBRC 13195), tunicamycin B1 (also present in the genome of *L. flaviverrucosa* As40578). Similar to the NRPS, the NRPS-like and the hybrid clusters have also been found which are associated with the products like WS9326 (present in the genome of *L. albidocapilata* subsp. *violacea* IMSNU 50388); lagunapyrone A/lagunapyrone B/lagunapyrone C, tyrobetaine, s56-p1 (present in the genome of *L. aerocolonigenes* NBRC 13195); oxalomycin B (present in genome of *L. flava* JCM 3296); BD-12, arsono-polyketide (present in the genome of *L. cavernae* CGMCC 4.7367); vazabitide A, marinacarboline A/marinacarboline B/marinacarboline C/marinacarboline D (*L. fradiae* CGMCC 4.3506); sanglifehrin A (present in the genome of *L. jiangxiensis*); griseorhodin A (present in genome of *L. xinjiangensis* CGMCC 4.3525); clarexpoxcin (present in the genome of *L. terrae* NEAU-LZS) (Dataset S1).

### Species‑specific putative products/analogues of known products

**Figure 10:**
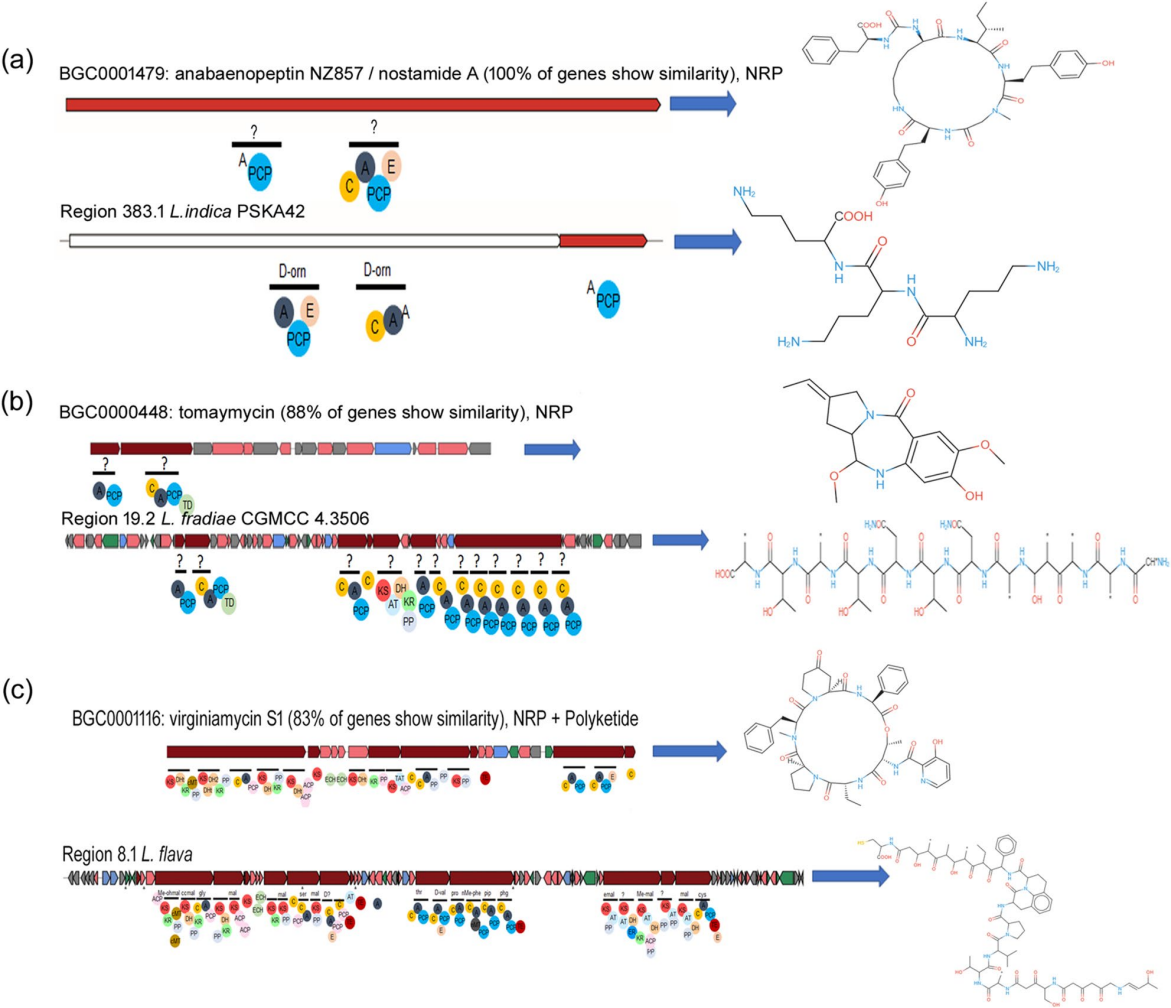
Species specific clusters and their putative products of *Lentzea* compared to the known clusters and their product from the antiSMASH database. (**a**) Biosynthetic cluster of anabaenopeptin NZ857/nostamide A of *Nostoc punctiforme* PCC 73102; (**b**) tomaymycin cluster of *Streptomyces achromogenes* compared with 88% similar genetic cluster of *L. fradiae* CGMCC 4.3506 and (**c**) genetic cluster for a macrolide group of antibiotics virginiamycin S1 of *L. flava* JCM 3296 having 83% similarity with that of *Streptomyces virginiae*.

Some BGCs have been identified which code for putative valuable compounds exclusively found in particular species. Although many similar products were already characterized from different sources but there are some compounds that are specific to *Lentzea* species. Here we have considered the clusters which have more than 50% similarity. The *L. indica* PSKA42 contains two regions, one is region 2.1 consisting of 41,241 nt (NRPS-like gene) but only a small number of genes is 100% similar to the known BGCs rhizomide A/rhizomide B/rhizomide C of *Paraburkholderia rhizoxinica* HKI 454^28^. But rhizomide A/rhizomide B/rhizomide C is having only one large ORF (22,977 nt) which is divided into seven modules, unlike the query genome which has only one. Another cluster showed 100% sequence similarity with a known cluster that is associated with the biosynthesis of anabaenopeptin NZ857/nostamide A of *Nostoc punctiforme* PCC 73102^29^. Although it showed 100% sequence similarity, the domain and structural organisation are completely different (Fig. 10a). Anabaenopeptin NZ857/nostamide A coded by one core NRPS gene (6546 nt) which comprised two modules (domain: A-PCP and C-A-PCP-E) in *Nostoc punctiforme* PCC 73,102 but *L. indica* PSKA42 contains two core NRPS genes (6,172 nt) which distributed in three modules (domain: A-PCP-E, C-A, and PCP). The lassopeptide achromosin is 100% similar to that of *L. jiangxiensis*. Tomaymycin (having antibiotic and antitumor activity) cluster of *Streptomyces achromogenes*^30^ shows 88% sequence similarity with the one present in *L. fradiae* CGMCC 4.3506. The *L. fradiae* genome contains a large cluster with a combination of PKS, PKS-like, NRPS and NRPS-like genes among which there are two core genes similar to *tomA* and *tomB* core genes of *S. achromogenes*, however, the rest of the structural organization are different (Fig. 10b). Putative polymer prediction of the *L. jiangxiensis* cluster is (X) + (X) + (X) + (pk) + (X − asn) + (thr − asn − thr − X − thr − X). The genetic cluster for virginiamycin S1, a macrolide group of antibiotics^31^, has been found in *L. flava* JCM 3296 (with 83% similarity). This antibiotic has been isolated from *Streptomyces virginiae* and the gene cluster of *Lentzea* is somewhat different in structure (Fig. 10c). Polymer prediction by *L. flava* genome is (ohmal − ccmal − gly) + (mal) + (mal) + (ser − mal) + (D-X) + (thr − D-val) + (pro − phe − pip − phg) + (emal − pk − Me-mal) + (pk) + (mal − cys). The mirubactin (siderophore compound) cluster shows 60% sequence similarity with the hybrid genetic cluster (T3PKS, T1PKS) of *L. californiensis* DSM 43393. Compound A-94964 is a nucleoside inhibitor active against phospho-N-acetylmuramyl-pentapeptide translocase which is required for bacterial peptidoglycan biosynthesis^32^. The genetic cluster for telomerase inhibitor griseorhodin A found in *L. albidocapilata* subsp. *albidocapillata* DSM 44,073 but the sequence similarity is low (63%) compared to the standard gene clusters. Antitumor antibiotic himastatin exhibits 60% identity with the hybrid cluster (NRPS-like, NRPS, other) of *L. flaviverrucosa* As40578. Some clusters show sequence similarity < 60% with the clusters of several products like tallysomycin A (anticancer), showdomycin (potent nucleoside antibiotic), iso-migrastatin/migrastatin/dorrigocin A/dorrigocin B/13-epi-dorrigocin A (inhibitors of tumor cell migration), candicidin (antifungal), pimaricin (antifungal) by the cluster of hybrids NRPS and T1PKS (*L. xinjiangensis* CGMCC 4.3525), ectoine (*L. nigeriaca* DSM 45680), transAT-PKS (*L. xinjiangensis* CGMCC 4.3525), hybrid T1PKS, NRPS (*L. kentuckyensis* NRRL B-24416), T1PKS (*L. indica* PSKA42) (Fig. S7).

### Ribosomally synthesized and post-translationally modified peptides (RiPPs)

As previously stated, all genomes contain 692 BGCs, of which 140 belong to RiPPs and their derivatives (thiopeptide, lanthipeptide, lassopeptide, LAP). These 140 clusters are distributed in RiPPs (77), RiPP-like (26) and RiPP hybrid clusters (37). However, just a few clusters (31) have been identified and recognized as putative products. All strains have a lanthipeptide-class-III gene that has 75% identity to that of the *Saccharopolyspora erythraea* NRRL 2338 product erythreapeptin-9 (Ery-9)^33^ (Fig. S8). Lanthipeptide has a wide range of bioactivities including antifungal, antimicrobial, and antiviral properties^34^. Ery-9 is a lantibiotic (specialized lanthipeptide compound) with antibacterial properties. Three species (*L. kentuckyensis* NRRL B-24416, *L. atacamensis* DSM 45479, *L. deserti* DSM 45480) contain lassopeptide encoding region which is known (100%) as citrulassin B isolated from *Streptomyces avermitilis* MA-4680. Genetic cluster for citrulassin D is found in *L. xinjiangensis* CGMCC 4.3525 and *L. flava* JCM 3296. The genome of *L. guizhouensis* DHS C013 contains genes for a lassopeptide, citrulassin F and this genetic region shows 100% similarity with *Streptomyces avermitilis* MA-4680 (Fig. S9). Lassopeptides could be used to treat tuberculosis, fungal infections, Alzheimer's disease, cardiovascular disease, cancer, and gastrointestinal illnesses^35^. This peptide ensures higher stability against heat, protease degradation and extreme pH^35^. Another cluster commonly thought to be a species-specific cluster, present in *L. jiangxiensis* CGMCC 4.6609 also codes for a lassopeptide antibacterial compound achromosin. Various low similarity (4–25%) putative RiPP like genetic clusters that code for 9-methylstreptimidone (ladderane, lassopeptide), glycinocin A (lassopeptide), lactazole (thiopeptide, LAP), SW-163C/UK-63598/SW-163E/SW-163F/SW-163G (lanthipeptide-class-I, other, NRPS), aborycin (lassopeptide) are found in *L. aerocolonigenes* NBRC 13195, *L. nigeriaca* DSM 45680, *L. pudingi* CGMCC 4.7319, *L. deserti* DSM 45480, *L. jiangxiensis* CGMCC 4.6609, and *L. terrae* NEAU-LZS genome. Thiopeptides are sulphur-rich macrocyclic peptides (contain extensively modified amino acids) antibiotics produced by bacteria and have activity against Gram-positive bacteria but little or no activity against Gram-negative bacteria^36^.

Additionally, PRISM analysis shows all genomes contain 418 of BGCs among them some important clusters are NRPS, PKS, PKS/NRPS, RiPP (thiopeptide, lanthipeptide, lassopeptide), angucycline-type polyketide, Kasugamycin family aminoglycoside contain 113, 80, 57, 95, 21, 5, respectively (Fig. S10).

### Phylogenetic analysis of the PKS KS domain, NRPS C domain

**Figure 11:**
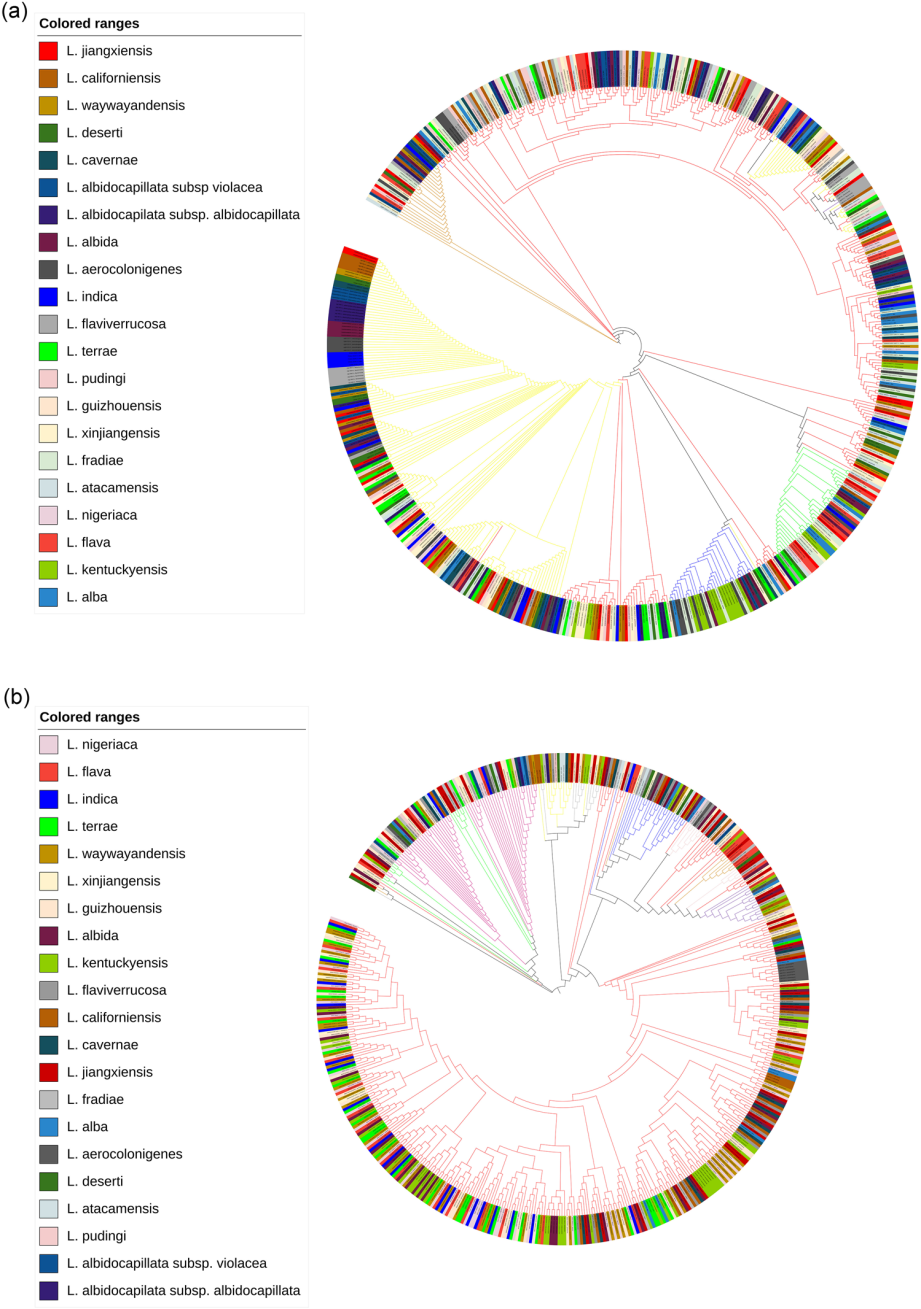
Phylogenetic analysis of (**a**) condensation and (**b**) ketosynthase domains of NRPS and PKS genes in *Lentzea* genomes by Neighbor-joining method against NaPDoS database domains. Leaves are coloured to represent different *Lentzea* species, while coloured branches display domain class of database domains (A) NRPS C domain: Yellow = DCL, Red = LCL, Green = cyc, Blue = modAA, Light Brown = start, Purple = C. (B) PKS KS domain: Yellow = enediyne, Red = modular, Green = FAS, Blue = hybrid KS, Brown = trans, Purple = KS1, Pink = typeII, Light pink = iterative.

Among the total 692 BGCs, 82.51% (total 571) and 75.72% (total 524) were from C domain and KS domain of NRPS and PKS genes, respectively (Fig. 11a, Dataset S2). The sequence similarity of all strains containing NRPS C domain ranges between 24 and 64%, except only one sequence of *L. xinjiangensis* CGMCC 4.3525 which has 78% similarity. Total six classes such as LCL, DCL, modAA, C, Starter (start), and heterocyclization (Cyc) were identified in the genomes. Among them, the most abundant is LCL class followed by the DCL type. The domain LCL and DCL are present in all species but C, Starter and Cyc are found only in fifth, sixteenth and seventeenth species, respectively. A LCL domain is responsible for the formation of peptide bonds between two L-amino acids and whereas DCL domain connects a L-amino acid to a developing peptide ending with a D-amino acid. Starter C domain acylates the first amino acid with a beta-hydroxycarboxylic acid and Cyc domains catalyze both peptide bond formation and subsequent cyclization of cysteine, serine or threonine residues. The modAA domain is engaged for modification of the incorporated amino acid^37^. These six classes were matched with pathways associated with the biosynthesis of microcystin (164), syringomycin (99), actinomycin (51), calcium-dependent antibiotic (49), bleomycin (32), cyclomarin (27), bacillibactin (21), bacitracin (19), complestatin (7), cyclosporin (7), fengycin (4), gramicidin (1), iturin (7), lychenicin (8), mycosubtilin (8), pksnrps2 (3), pristinamycin (12), pyochelin (8), thiocoraline (6), tyrocidine (17), yersiniabactin (11) sporolide (1), and surfactin (6) but with very low sequence similarity (average of 41%) indicating their association with different compounds. Functional classification depicted that total 524 KS domains present in each of the *Lentzea* genomes which are distributed in nine classes, among them most are from modular class and the rest of them are trans, PUFA, KS1, hybrid KS, iterative, FAS, and enediyne. Two types T1PKS are found—modular and iterative. The modular PKS enzymes are large multi-domain enzymes that only utilize each domain once during the synthesis process whereas the iterative PKS use the same domain numerous times^38^. Out of 21 species, 5 species (*L. albidocapillata* subsp *violacea* IMSNU 50388, *L. albidocapilata* subsp. *albidocapillata* DSM 44073, *L. deserti* DSM 45480, *L. atacamensis* DSM 45479, and *L. pudingi* CGMCC 4.7319) contain only 3–7 KS domain. The similarity among KS domains of all strains ranges between 25 and 84% (except one sequence of *L. albida* DSM 44437; 85%) with an average similarity of 66.39%. The sequence of this class are similar to genes associated with various biosynthetic pathways which lead to the formation of nystatin (182), avermectin (79), alnumycin (51), epothilone (46), aclacinomycin (6), alkylresorcinol (5), PUFA (8), trans (10), avilamycin (1), bleomycin (1), C-1027 (2), calicheamicin (9), esperamicin (5), fatty acid synthesis (15), heat-stable antifungal factor (HSAF, 2), leinamycin (4), maduropeptin (2), neocarzinostatin (1), rapamycin (10), rifamycin (2), saquayamycin (4), tetronomycin (67), tylosin (12), unknown (2), and virginiamycin (6). Although various strains, despite being belonged to different phylogenetic clades, are found to be able to synthesize the same kind of compounds. In other words, the same product forming pathways are found in multiple species but in phylogenetic analysis clustered them differently based on the sequence similarity (Fig. 11b, Dataset S2). In conclusion, accurate structural class of the compounds can be expected from these domains if the sequence similarity is > 90% with the biosynthetic domains of experimentally validated compounds^39^. But in this analysis, these domains could not fulfill the above criteria and thus clearly indicates that they are possibly the reservoir for new biomolecules.

### Distribution of CAZY enzymes

**Figure 12:**
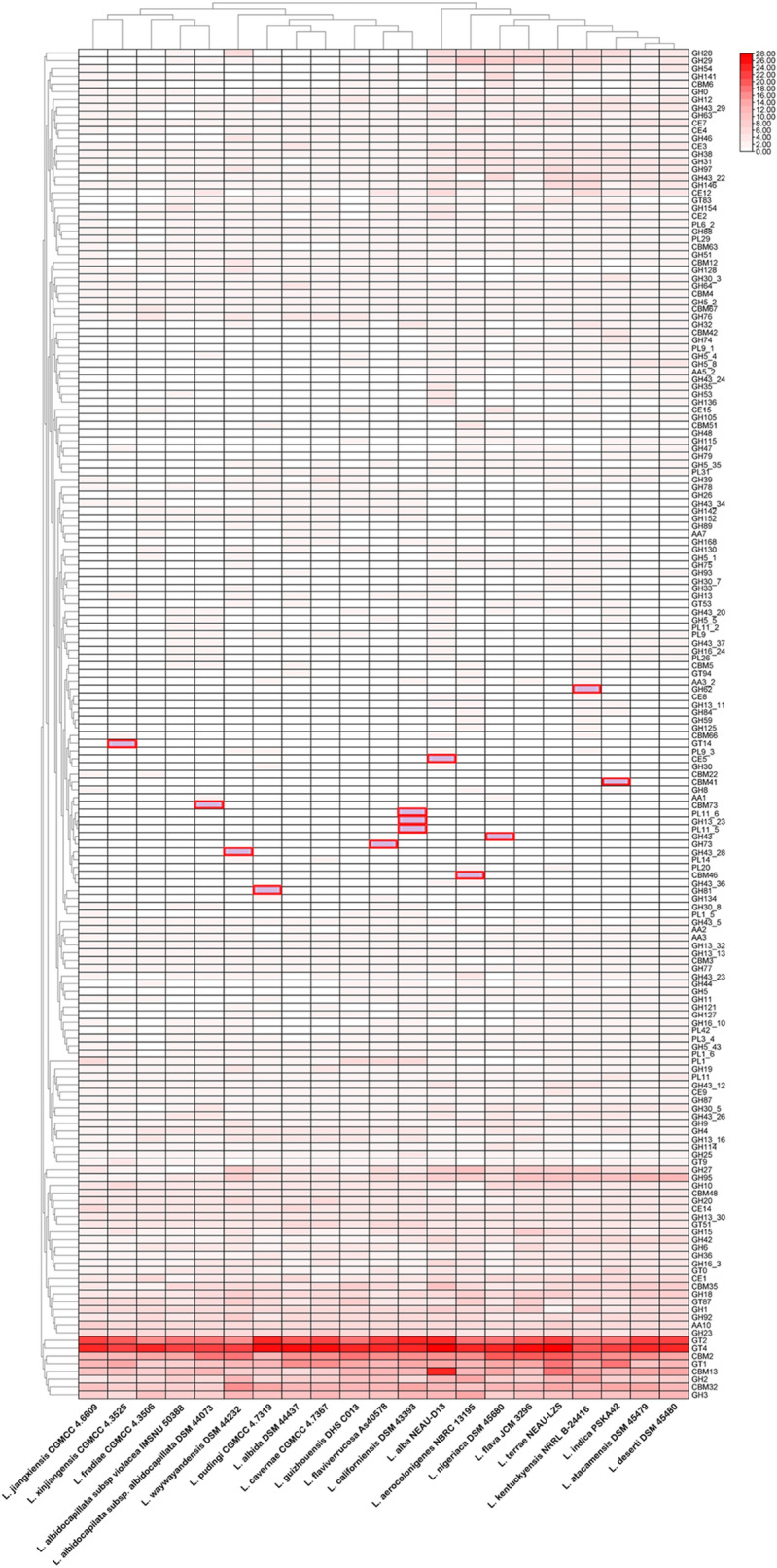
Heat map represents the abundance of the CAZymes present in *Lentzea* genome. The red color intensity is proportional to the abundance of the genes for CAZymes. Red box = unique enzymes.

**Figure 13:**
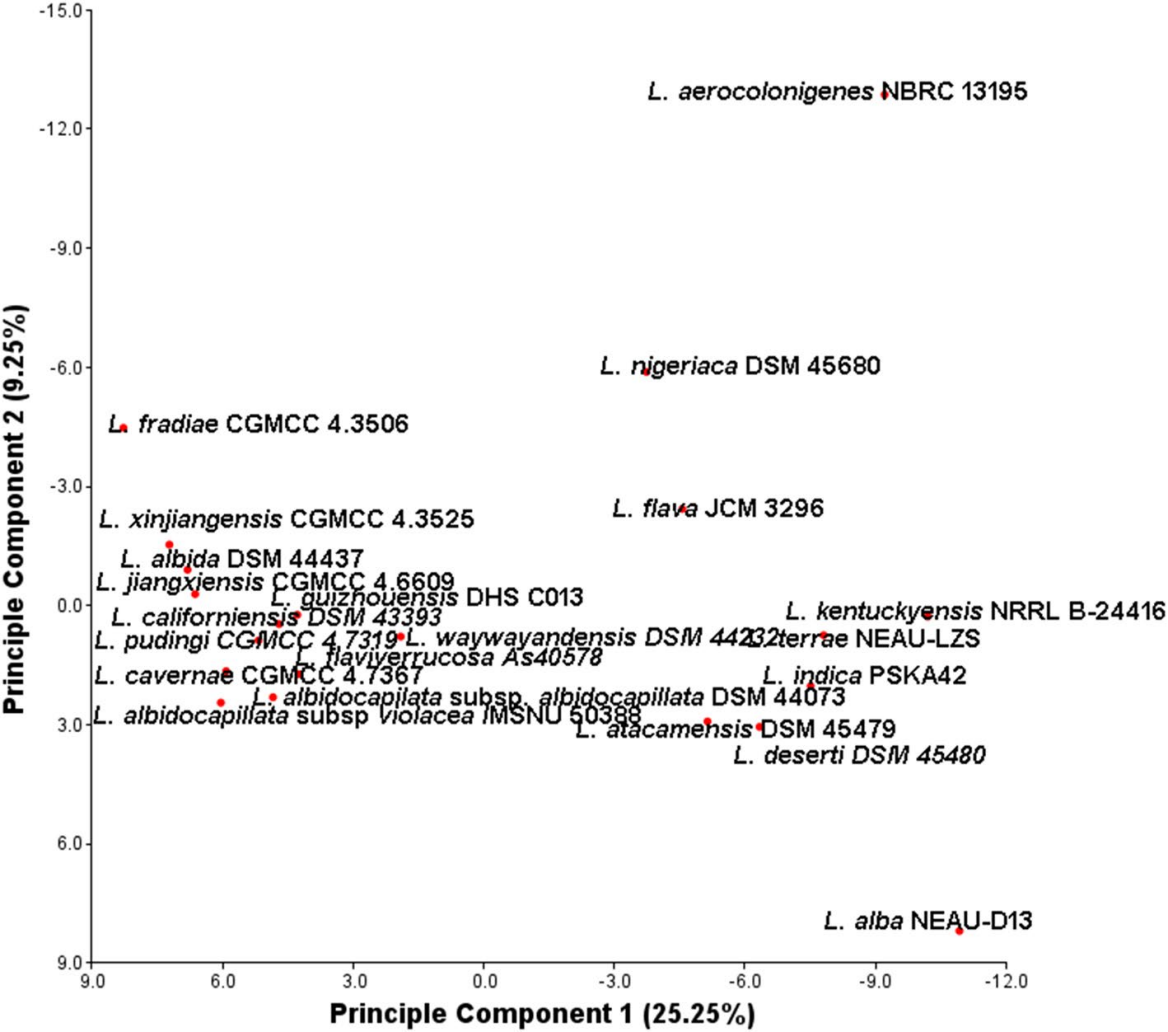
Principal component analysis (PCA) among the different *Lentzea* based on CAZY enzymes recovered from dbCN2 for their relationship.

Total of 7121 genes are involved for the production of CAZY enzymes and these are distributed in six families including families of auxiliary activities (AA), carbohydrate-binding modules (CBM), carbohydrate esterases (CE), glycoside hydrolases (GH), glycosyl transferases (GT), polysaccharide lyases (PL) in all *Lentzea* sp. (Fig. S11). GH is the source of the bulk of enzymes under these families followed by GT and CBM subsequently. Again, each family is distributed in various types and subtypes. Out of 7121 genes, the highest (428) and lowest (245) number of CAZymes are represented by *L. alba* NEAU-D13 and *L. fradiae* CGMCC 4.3506, respectively. In this study, we have found total of 190 various categories of the major six families CAZymes present in *Lentzea* genomes. We have identified some enzymes which are species-specific (Fig. 12). These are CBM41 (activity for α-glucans amylose, amylopectin, pullulan, and oligosaccharide fragments), CBM46 (cellulose), CBM73 (chitin-binding function), CE5 (acetyl xylan esterase, cutinase), GH13_23 (glucosyltransferase, oligo-a-1,6-glucosidase, glucosidase), GH43 (β-xylosidase; α-L-arabinofuranosidase; xylanase); GH43_28 (no known activity), GH62 (L-arabinofuranosidase), GH73 (lysozyme, mannosyl-glycoprotein endo-β-N-acetylglucosaminidase peptidoglycan hydrolase with endo-β-N-acetylglucosaminidase specificity), GH81 (endo-β-1,3-glucanase), GT14 (β-1,3-galactosyl-O-glycosyl-glycoprotein β-1,6-N-acetylglucosaminyltransferase, N-acetyllactosaminide β-1,6-N-acetylglucosaminyltransferase), PL11_5 (no known activity), PL11_6, PL_14 in *L. fradiae* CGMCC 4.3506, *L. indica* PSKA42, *L. aerocolonigenes* NBRC 13195, *L. albidocapilata* subsp. *albidocapillata* DSM 44073, *L. alba* NEAU-D13, *L. californiensis* DSM 43393, *L. nigeriaca* DSM 45680, *L. waywayandensis* DSM 44232, *L. kentuckyensis* NRRL B-24416, *L. flaviverrucosa* As40578, *L. pudingi* CGMCC 4.7319, *L. xinjiangensis* CGMCC 4.3525, *L. californiensis* DSM 43393, and *L. californiensis* DSM 43393, respectively. Similarly, four CAZymes such as CBM67 (L-rhamnose), GH5_43 (glucosidase), GH27 (α-galactosidase), and GH30_5 (endo-b-1,6-galactanase) are present in all strains except *L. jiangxiensis* CGMCC 4.6609, *L. fradiae* CGMCC 4.3506, *L. albidocapilata* subsp. *violacea* IMSNU 50388, and *L. fradiae* CGMCC 4.3506. Furthermore, heatmap also implies the differentiation/relationship among the *Lentzea* sp. (Fig. 12). Out of 190, the copy number of 16 different families of enzymes are same in all species. These are CBM16, CBM56, GH5_18, GH5_51, GH13_3, GH13_10, GH13_26, GH57, GH65, GH85, GH171, GT20, GT28, GT35, GT39, and GT81. Furthermore, 51 different families of enzymes such as AA10, CBM2, CBM4, CBM13, CBM32, CBM35, CBM48, CE1, CE3, CE7, CE9, GH9, CE14, GH1, GH2, GH3, GH4, GH6, GH10, GH12, GH13_13, GH13_16, GH13_30, GH13_32, GH15, GH16_3, GH18, GH19, GH20, GH23, GH25, GH36, GH38, GH42, GH43_12, GH43_26, GH64, GH76, GH77, GH87, GH92, GH95, GH97, GH146, GT0, GT1, GT2, GT4, GT51, GT87 and PL11 are common in all *Lentzea* species however the copy numbers are different in different species (Fig. 12). The PCA also shows their similarity in CAZymes, which is found consistent with their cladogram (Fig. 13). The prediction of different signal peptide variants in specific enzymes exhibited that it is either freely secreted or retained in the cell. Some examples of these enzymes are GT51, GH23, GH28, CBM13, CBM32, CBM35, etc. (Dataset S3). On the contrary, there are enzymes such as CE9, CE14, GT1, GT2, GH42, GT28, GT39, etc. found in this study (Dataset S3) which are devoid of any predicted signal peptide and thought to be anchored to the cell. Similarly, some enzymes are also found which is freely secreted by the presence of signal peptide such as AA10, PL29, PL42, GT35, etc. (Dataset S3).

## Discussion

This comparative genomics of *Lentzea* species deals with the genome annotations by the several bioinformatics approaches such as RAST, KEGG, webMGA, antiSMASH, PRISM, NaPDos, and dbCN2 for the identification of genetic clusters that are responsible for coding bioactive compounds of medicinal and biotechnological relevance. Most of the genes, as found from RAST annotation, are involved in the metabolism of amino acids, carbohydrates and cofactors, vitamins, and prosthetic groups. COGs proteins related to the general function, transcription, carbohydrate and amino acid transport and metabolism are having most abundance in this genus. Similarly, proteins identified through KEGG are predominantly associated with metabolism purposes. In a nutshell majority of *Lentzea* genes are participate in various metabolic functions. Genome-based comparison through the ANI and dDDH values dictate that all species are distinctly different from each other, however, two species (*L. atacamensis* DSM 45479 and *L. deserti* DSM 45480) should be classified in a single species. The phylogenomic study also reveals that they are well separated but the branch length of two species (*L. atacamensis* DSM 45479 and *L. deserti* DSM 45480) are the same which further indicates that they could be identical species. Core protein-based phylogenetic analysis also supports the same observation as WGS-based phylogenetic analysis of the above two species. This study insight into the bioactive and CAZY enzymes producing genes across the 21 *Lentzea* species of actinobacteria. Although the genome sizes are larger in some species, the number of BGCs is not increased proportionally, i.e., the number of BGCs is similar amongst the species. The top order of identified BGCs, as unraveled by antiSMASH, are 185 hybrids (NRPS, PKS, terpene, RiPP and others), 124 terpene, 116 others (arylpolyene, indole, CDPS, NAPAA, betalactone, etc.), 77 RiPP, 74 PKS (T1PKS, T2PKS, T3PKS and hglE-KS), 37 NRPS, 36 NRPS-like, 26 RiPP-like and 17 are for siderophore. Almost all genomes exhibit at least a single cluster of T1PKS genes (except *L. pudingi* CGMCC 4.7319 and *L. flava* JCM 3296), NRPS (except *L. albida* and *L. flaviverrucosa*), NRPS-like (except *L. kentuckyensis* NRRL B-24416) and lanthipeptide-class-ii (except *L. indica* PSKA42, *L. nigeriaca* DSM 45680). *L. indica* PSKA42 showed the most abundant clusters of T1PKS, NRPS and NRPS-like compared to all other species. We have identified highly similar clusters (> 50%) present in the *Lentzea* genome that are similar to the chemically characterized well-known compounds and are in use for various purposes (Fig. S12). These products are rhizomide, bezastatin derivates (NRPS-like); coelichelin, anabaenopeptin/NZ857/nostamide A, indigoidine, amychelin (NRPS); Ery-9, citrulassin F, citrulassin B (RiPP); geosmin, isorenieratene, 2-methylisoborneol (terpene); BE-54017, staurosporine (indole), vancosamine (arylpolyene); cyclizidine, A-94964, himastatin, LLD, virginiamycin S1, tomaymycin, nystatin A1, nystatin, mirubactin (hybrid); pimaricin, butyrolactol A (PKST1); alkylresorcinol, alkyl-O-dihydrogeranyl-methoxyhydroquinones (PKST3); griseorhodin A(PKST2); iso-migrastatin (transAT-PKS) and showdomycin (ectoine). Some of these above compounds are species-specific (Fig. S12). Furthermore, the *Lentzea* BGCs clusters with less than 50% sequence similarity are the potential repertoire of wide ranges of antibiotics. Among these, some belong to the new generation antibiotics such as calcium-dependent, terpenoid, glycopeptide, lassopetide antibiotics. Calcium-dependent antibiotics need calcium ions for their function and generally target the cell membrane. Daptomycin is the ideal example of such antibiotics that works against methicillin-resistant *Staphylococcus aureus*^40^. Terpenoid antibiotics are usually oil substances that seem to disrupt two critical microbial survival processes: oxygen absorption and oxidative phosphorylation^41^. As stated earlier, *Lentzea* genomes are the reservoirs of many antitumor compounds among which showed very low similarity with the known anti-tumour compounds of bacterial origin such as bleomycin and actinomycin D used in case of Wilms tumour, Hodgkin’s lymphoma, non-Hodgkin’s lymphoma, Ewing’s sarcoma, trophoblastic neoplasm, testicular cancer, cervical cancer and ovarian cancer. Several genetic clusters that code for antivirals, insecticidal and antiparasitic compounds are also found. Among them, clusters for compounds like echinomycin (antibacterial, anticancer, and antiviral activities) and paromomycin (for parasitic infections including amebiasis, giardiasis, leishmaniasis, and tapeworm infection) are very common. It clearly highlights that all species of *Lentzea* may have the potentiality to provide future antibacterial compounds by virtue of having the genetic clusters that code them. Also, we have identified 5 species that contain insecticidal/antiparasitic known clusters, among them *L. waywayandensis* DSM 44,232 is having mostly abundant clusters for the production of such compounds. Similarly, antimycobacterial and antiviral compound-producing genetic clusters are found in 7 and 8 species, respectively. Out of 21 species, 18 showed gene clusters for antitumor and antifungal compounds (Table 2). However, the 190 BGCs (out of 692) are unable to match with any of the known products which seem to be cryptic gene clusters^42^, implying that *Lentzea* sp. could be a viable source for novel bioactive discoveries. To our knowledge this genus of actinobacteria was not used extensively for exploration of the above bioactive compounds, therefore, more study will be needed for extraction of compounds and their characterization or activation of cryptic genes, if it will not work, in invitro. Other than bioactive compounds, these bacteria can be used in agricultural and environmental sectors for having genes for siderophores production, and enzymes (AA, CBM, CE, GH, GT, PL) that convert several carbohydrates like chitin, pectin, xylan, cellulose, starch, lactose, maltose, arabinose, xylose, etc. The GH is responsible for the hydrolysis of glycosidic bonds present in the complex sugars (cellulose, hemicellulose, starch, lactose, maltose, chitin, sucrose, xylan) and used in defense strategies such as lysozyme, viral neuraminidases^43^. On the other hand, the GT has been recognized for forming natural glycosidic connections and is widely exploited in the targeted synthesis of certain glycoconjugates or the development of glycosylated mediated drugs^44^. The CBM is an important protein module of cellulases, especially cellobiohydrolases^45^. The presence of acetylated glycosyl residues in plant polysaccharides (O-acetylated) protects the plant cell wall from microbial attack by preventing glycoside hydrolases mediated breakdown of glycosidic bonds. By removing the acylated moieties of polysaccharides, easy to access of GHs to accelerate their breakdown and CE can help in biomass saccharification for biofuel production. CE can undergo the breakdown of plant polysaccharide de-acetylate hemicellulose and pectin units^46^. PLs are a group of enzymes that cleave uronic acid-containing polysaccharides (pectins, alginates and heparins) therefore it has huge application in the food and medical sectors^47^. The largest component of the plant cell wall is lignocelluloses, which comprises three components such as cellulose (40–50%), hemicellulose (25–30%), and lignin (15–20%)^48^. Based on function, cellulase is three types- endoglucanase, exoglucanase, and β-glucosidase. These three enzymes containing genes are identified by dbCAN2 meta server for the prediction of CAZyme such as GH5, GH6, GH9 and GH12 (endoglucanase), GH6, GH9 and GH48 (exoglucanase) and GH1 and GH3(β-glucosidase) which are found in *Lentzea* sp. and are similar with other genera^48,49^. Hemicellulose is comprised of a polymer of different sugars such as mannose, xylose, glucose, galactose and arabinose. Hemicellulases are a group of enzymes involved in the breakdown and hydrolysis of mannans, galactans, xylans, and arabans. Xylan degradation enzymes are endo-1,4-β-xylanase, β-xylosidase, α-arabinofuranosidase, acetyl xylan esterase and polysaccharide deactylase. Xylan degrading genes associated with mainly with GH families such as 5, 7, 8, 9, 10, 11, 12, 16, 26, 30, 43, 44, 51, and 62^48^. All of these GH families are found in *Lentzea* such as endo-1,4-β-xylanase (GH5, GH8, GH10, GH11, GH30, GH43, GH48), β-xylosidase (GH39, and GH43), α-arabinofuranosidase (GH12, GH43, GH51, and GH62) and other xylanase is acetyl xylan esterase (CE1, CE3 and CE7) and polysaccharide deacetylase (CE4), respectively. Mannan (polymer of mannose) degradation is also associated with GH families’ enzymes also found in the present study such as α-mannosidase (GH38), α-1,6-mannanase (GH76) β-mannanase (GH5, GH26), β-mannosidase (GH2) and α-galactosidase (GH27). Two enzymes such as GH18 and auxiliary activity families (AA10, AA11 and AA15) are the most significant enzymes for chitin degradation although GH19 is well known but it has less active against crystalline chitin^50^. The chitin degradation enzymes present in *Lentzea* sp. and also reported in many studies^50–52^ such as chitinase (GH18, GH19, GH23, GH48), lytic cellulose monooxygenase (AA10), N-acetylglucosaminidase (GH20), chitin synthase (GT2), chitosanase (GH5, GH8, GH46, and GH75), chitin deacetylase (CE4) and some CBM (CBM3, CBM5, CBM12) families. Furthermore, other different GH (including some GH family of above mentioned) annotated genes related to polymer degradation are found to be coupled with carbohydrate-binding modules (Dataset S3). Decolouration of the textile dye takes place enzymatically by laccases, ligninolytic peroxidases or cytochrome P450 monooxygenases. We have found genes coding ligninolytic enzymes belong to two laccases family AA1 and AA2^53^. Biofuel production (such as bioethanol) from plants biomass often is troublesome due to the presence of excessive lignin but the above-mentioned microbial enzymes (AA) could be an alternative candidate to overcome the issue^54^. These actinobacteria possess some CE families which are potential targets for drug design such as CE1, CE4, CE7, CE and CE14^55^. Degradation/utilization of these carbohydrates were also reported in invitro study by these bacteria^16,17,56^.

**Table 2:** Number of putative genetic clusters for bioactive compounds present in various *Lentzea* genome.

Organisms	Anti-bacterial	Antitumor	Anti-mycobacterial	Antifungal	Antiviral	Insecticidal/antiparasitic
*L. indica* PSKA42	5	1	1	3	–	–
*L. guizhouensis* DHS C013	8	4	1	2	–	–
*L. albidocapillata* subsp. *violacea* IMSNU 50388	2	1	1	–	1	–
*L. albidocapilata* subsp. *albidocapillata* DSM 44073	5	4	–	–	–	–
*L. aerocolonigenes* NBRC 13195	2	–	1	1	–	–
*L. californiensis* DSM 43393	3	3	–	1	–	–
*L. flaviverrucosa* As40578	1	3	–	1	–	–
*L. nigeriaca* DSM 45680	3	3	–	2	1	–
*L. atacamensis* DSM 45479	3	1	–	1	–	–
*L. pudingi* CGMCC 4.7319	6	2	1	–	–	–
*L. flava* JCM 3296	3	2		1	1	–
*L. cavernae* CGMCC 4.7367	4	3	1	1	1	–
*L. deserti* DSM 45480	2	2	–	1	–	–
*L. waywayandensis* DSM 44232	4	4	–	3	1	1
*L. albida* DSM 44437	4	–	–	3	–	1
*L. fradiae* CGMCC 4.3506	5	–	–	3	1	1
*L. jiangxiensis* CGMCC 4.6609	4	3	–	1	–	–
*L. alba* NEAU-D13	2	–	–	–	–	–
*L. xinjiangensis* CGMCC 4.3525	5	1	–	2	1	3
*L. kentuckyensis* NRRL B-24416	3	2	–	1	–	1
*L. terrae* NEAU-LZS	1	2	1	3	2	–

## Conclusion

**Figure 14:**
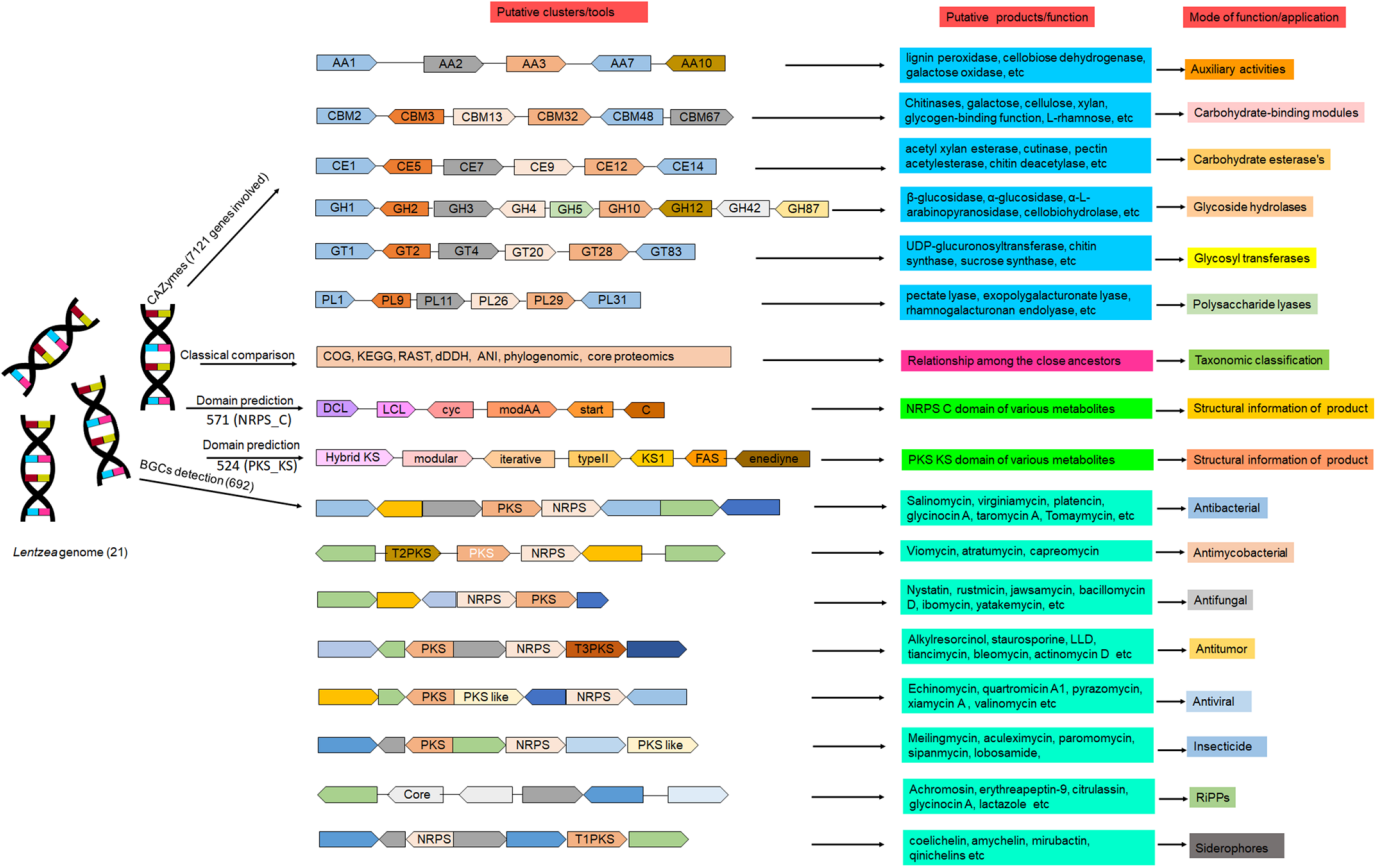
Schematic representation of the summarized work flow for *Lentzea* genome analysis to demarcate the gene clusters responsible for the production of CAZymes and pharmaceutically relevant secondary metabolites.

In summary, we described the genomic features, phylogenomic, core-proteomics based phylogenetic analysis, various secondary metabolite producing genes and their putative products, polymer degrading ability enzymes of all *Lentzea* species (Fig. 14). All genomes contain some common clusters such as terpene, RiPP (lanthipeptide-class-iii), RiPP-like, redox-cofactor, and NAPAA which indicates that these clusters are conserved in this bacterium. With the wide varieties of BGCs, this rare species of actinobacteria have potential to make unique and versatile putative products. Considering these in mind, it can be expected that such a genus will be used for the synthesis of various bioactive compounds including antitumor, antibiotics, antiviral, antifungal, anthelminthic, and insecticide (Fig. 14). This work may help to provide target-specific products which are only found in particular species. Furthermore, *Lentzea* may be used in the agricultural sector due to the degrading ability of various polymers and fulfilment of various nutritional requirements by the crop plant.

## Methods

### *Lentzea* whole genome-sequence retrieval

The genome sequence of all available type strains of *Lentzea* (a total of 21 including strain PSKA42) has been accessed and downloaded from the public database National Center for Biotechnology Information (NCBI) and EzBioCloud database. Different annotation programs have different results (contig number, genome size, L50, N50, % GC content, number of genes, number of protein-coding genes) therefore, all genomes were subjected to annotation only by using the Rapid Annotation utilizing Subsystem Technology (RAST) server^57^, and the genomic properties were described in details (Table 1, Table S1).

### Comparison of the available genome sequences

All the annotated protein sequences were evaluated for clusters of orthologous groups (COGs) using WebMGA (http://weizhong-lab.ucsd.edu/webMGA/) which imparts the functional annotation and phylogenetic relationship^58^. Functional annotation has also been performed by KEGG database which assigns an ortholog group known as KEGG Orthology (KO) to each taxonomic category by internally used KOALA algorithm after the BLAST from all the available full sequenced genomes^59^. Phylogenomic of strain PSKA42^T^ was carried out by TYGS (Type strain genome server) in order to predict the relationship with all species of *Lentzea*. The open reading frames (ORFs), GC content, and orthologous groups from each genomic sequence were automatically executed based on M1CR0B1AL1Z3R pipeline (https://github.com/orenavram/microbializer) with the default parameters as described by Avram et al.^60^. Phylogenetic analysis of core proteomes was performed by MEGA7.05. The dDDH (digital DNA-DNA hybridization) values were calculated using the recommended settings of the GGDC 2.1 from genomes of all *Lentzea* species^19^. The JSpecies WS was used to calculate the average nucleotide identity (ANI) of all species using the ANI-Blast (ANIb) and ANI-MUMmer (ANIm) algorithms^20^. All genomes were aligned and compared by BLAST Ring Image Generator (BRIG) to create a circular map of each genome^61^.

### Identification of BGCs, PKS KS domain, NRPS C domain

The BGCs present in the genomes of *Lentzea* were identified using antibiotics and secondary metabolite analysis shell (antiSMASH v5.0.0) (https://antismash.secondarymetabolites.org)^62^, and PRediction Informatics for Secondary Metabolomes (PRISM)^63^. For phylogenetic analysis of both KS and C domains of PKS and NRPS genes, respectively, were performed after retrieving the relevant sequences from all identified BGCs. For functional classification, the query sequences were compiled in one FASTA file and analyzed through Natural Product Domain Seeker (NaPDoS)^39^ database. All sequences were trimmed in equal length and the phylogenetic tree was constructed by MEGA7.05 followed by editing in iTOL v6^64^.

### Prediction of CAZY enzymes

All genomes were analyzed for the identification of genes that codes carbohydrate-active enzyme (CAZymes) using dbCAN2 meta-server which includes HMMER, DIAMOND and Hotpep tools. An enzyme was considered for true annotation when it hits at least two tools out of three. Additionally, the signal peptides were detected by dbCAN2 of respective CAZymes in all genomes^65^.

### Statistical analyses

For heatmaps, hierarchical clustering of the different features of BGCs/CAZymes (columns) and the different samples/species (rows) were performed using the euclidean distance metric using the respective data (copy numbers of each subfamily of CAZymes and individual BGCs). Principal component analyses (PCA) were performed of all species for their relationship based on BGCs composition and CAZymes distribution using the respective data.

### Ethical approval

The research is not associated with any prior ethical approval.

### Consent of publication

The data used for the manuscript is original and does not require any consent from third party for publication.

## Supplementary Information

Supplementary Information 1.

Supplementary Information 2.

Supplementary Information 3.

Supplementary Information 4.

## Data Availability

All the data associated with the research are available as supplementary files.

## Abbreviations

antiSMASH: Antibiotics and secondary metabolite analysis shell
PRISM: PRediction informatics for secondary metabolomes
NaPDoS: Natural product domain seeker
BGCs: Biosynthetic gene clusters
RiPPs: Ribosomally synthesized and post-translationally modified peptides
CAZymes: Carbohydrate-active enzyme
RAST: Rapid annotation utilising subsystem technology
COG: Clusters of orthologous groups
KEGG: Kyoto encyclopedia of genes and genomes
BRIG: BLAST ring image generator
dDDH: Digital DNA-DNA hybridization
ANIm: ANI-MUM mer
ANIb: ANI-Blast
TYGS: Type strain genome server
NRPS: Non ribosomal polypeptide synthetases
PKS: Polyketide synthase
PCA: Principal component analyses
