# Supplementary material for: Comprehensive genome analysis of Lentzea reveals repertoire of polymer-degrading enzymes and bioactive compounds with clinical relevance
Source: Sci Rep. 2022 May 19;12:8409. doi: 10.1038/s41598-022-12427-7 (PMC9120177; doi:10.1038/s41598-022-12427-7)
Supplement: Supplementary file 4 — Supplementary Information 4. [file 41598_2022_12427_MOESM4_ESM.pdf]

Title: Comprehensive Genome Analysis of *Lentzea* Reveals Repertoire of Polymer-degrading Enzymes and Bioactive Compounds with Clinical Relevance

Author: Pulak Kumar Maiti*, Sukhendu Mandal*

Affiliations:

Laboratory of Molecular Bacteriology, Department of Microbiology, University of Calcutta, 35, Ballygunge Circular Road, Kolkata, 700019, India

***Corresponding Author:** Sukhendu Mandal

Address: Laboratory of Molecular Bacteriology, Department of Microbiology, University of Calcutta, 35, Ballygunge Circular Road, Kolkata, 700019, India.

Email: sukhendu1@hotmail.com

ORCID ID: https://orcid.org/0000-0002-7752-0982

***Co-corresponding Author:** Pulak Kumar Maiti

Address: Laboratory of Molecular Bacteriology, Department of Microbiology, University of Calcutta, 35, Ballygunge Circular Road, Kolkata, 700019, India.

Email: pulak.micro@gmail.com

ORCID ID: https://orcid.org/0000-0001-7422-0960

Table S1: NCBI Prokaryotic Genome Annotation Pipeline (PGAP) of *Lentzea* sp.

**Organisms**	**Scaffolds/ contigs**	**N50/L50**	**GC%**	**Protein**	**rRNA**	**tRNA**	**Other RNA**	**Gene**	**Pseudo -gene**
L. indica PSKA42	634/637	30,279/94	68.3	8,896	14	61	3	9,967	993
L. guizhouensis DHS C013	1/1	9,997,872/1	70.0	9,215	15	73	3	9,721	415
L. albidocapillata subsp *violacea* IMSNU 50388	57/58	331,077/8	69.0	8,179	17	68	3	8,381	114
L. aerocolonigenes NBRC 13195	55/61	348,916/12	68.9	9,760	9	68	3	9,962	122
L. albidocapilata subsp. *albidocapillata* DSM 43393	37/43	397,314/9	68.7	8,108	14	62	3	8,284	97
L. californiensis DSM 43393*	34/ND	528,830	69.3%	8539	18	69	ND	8,630	ND
L. flaviverrucosa As40578	31/38	462,737/7	69.2	8,832	6	69	3	9,009	99
L. jiangxiensis CGMCC 4.6609	61/62	279,756/7	70.2	7,889	12	68	3	8,117	145
L. xinjiangensis CGMCC 4.3525	54/61	283,838/10	70.7	8,041	14	62	3	8,265	145
L. cavernae CGMCC 4.7367	39/43	472,751/7	69.6	9,120	5	64	3	9,298	106
L. waywayandensis DSM 44232	33/39	475,994/6	68.9	9,180	15	68	3	9,416	150
L. atacamensis DSM 45479	32/38	785,641/4	68.9	8,851	8	69	3	9,094	163
L. fradiae CGMCC 4.3506	43/47	479,571/8	70.5	7,898	15	62	3	8,071	93
L. pudingi CGMCC 4.7319	56/63	432,100/6	69.1	8,539	5	69	3	8,787	171
L. albida DSM 44437	40/43	352,714/9	70.2	8,716	15	65	3	8,883	84
L. terrae NEAU-LZS	59/145	211,542/17	68.7	9,722	6	67	3	10,001	203
L. kentuckyensis NRRL B-24416	317/ND	92,128/34	68.8	9,161	5	63	3	9,449	217
L. deserti DSM 45480	41/50	442,485/7	68.8	8,995	8	68	3	9,238	164
L. flava JCM 3296	188/190	133,734/20	69.0	8,918	7	71	3	9,207	208
L. nigeriaca DSM 45680	42/42	641,913/6	68.9	8,645	8	68	3	8,873	149
L. alba NEAU-D13	35/42	431,430/8	68.7	9,085	12	67	3	9,476	309

*Data from GOLD database

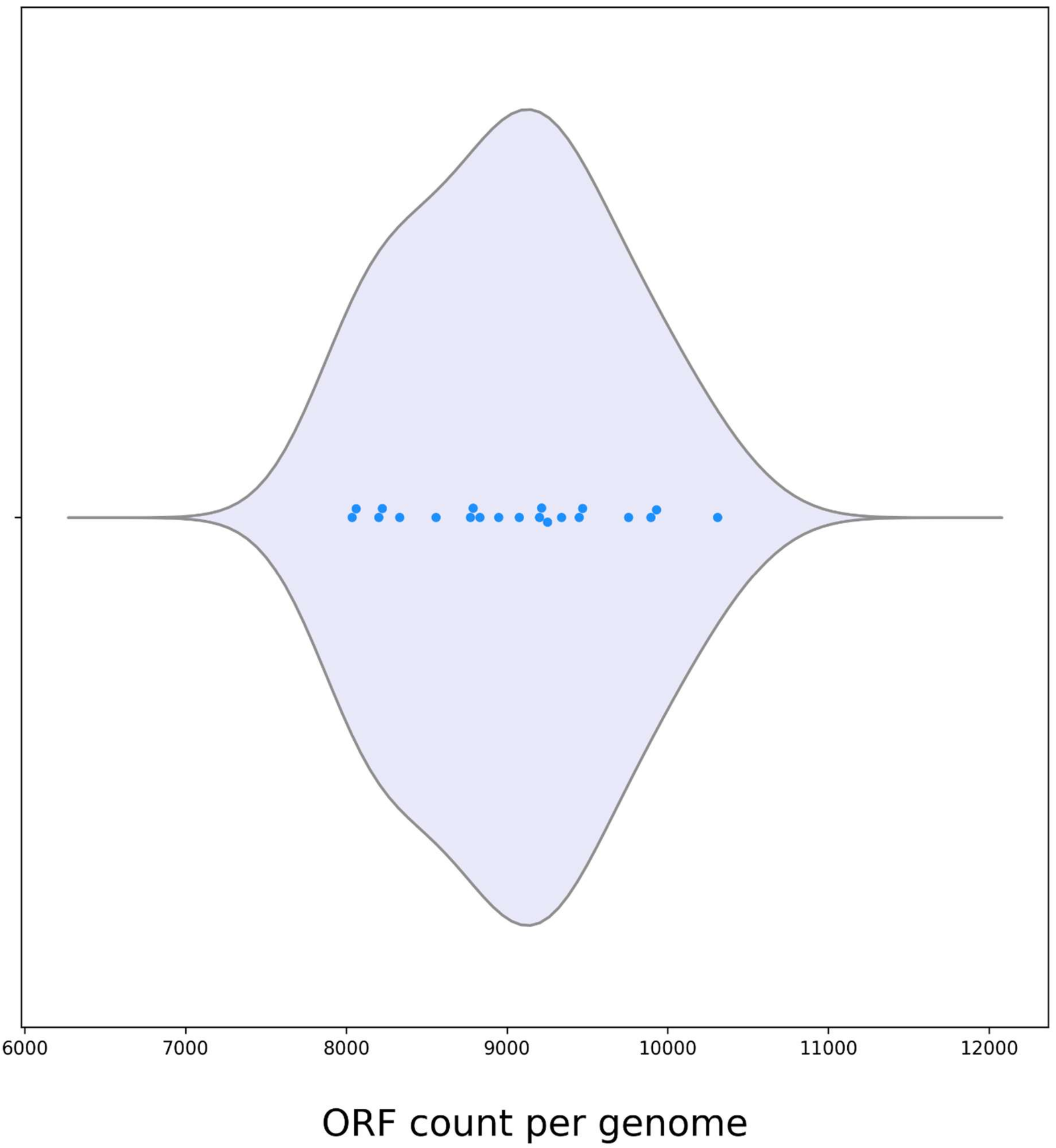

Fig. S1. ORF distribution in all *Lentzea* genomes

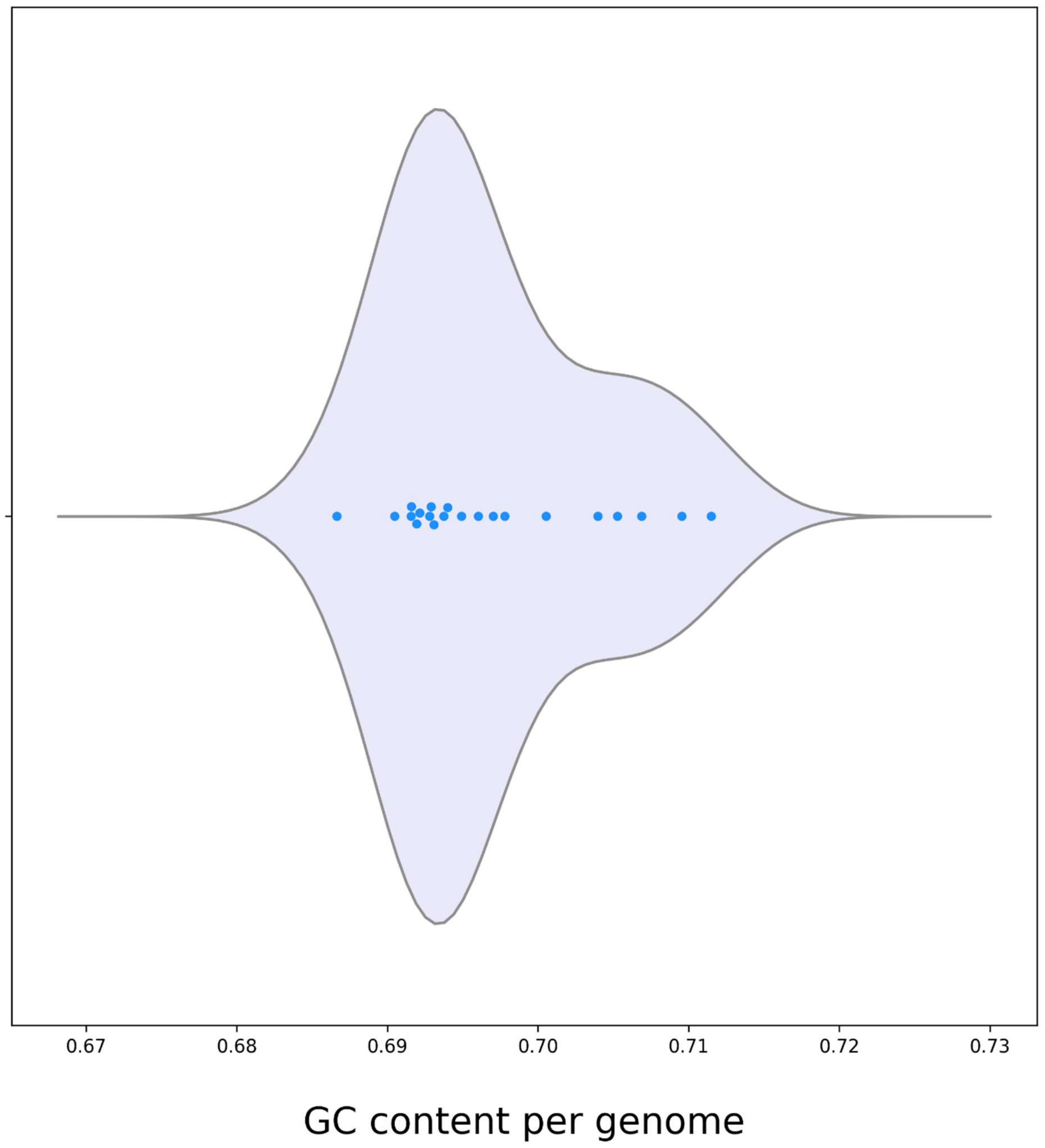

Fig. S2. GC content in each genome of *Lentzea* species

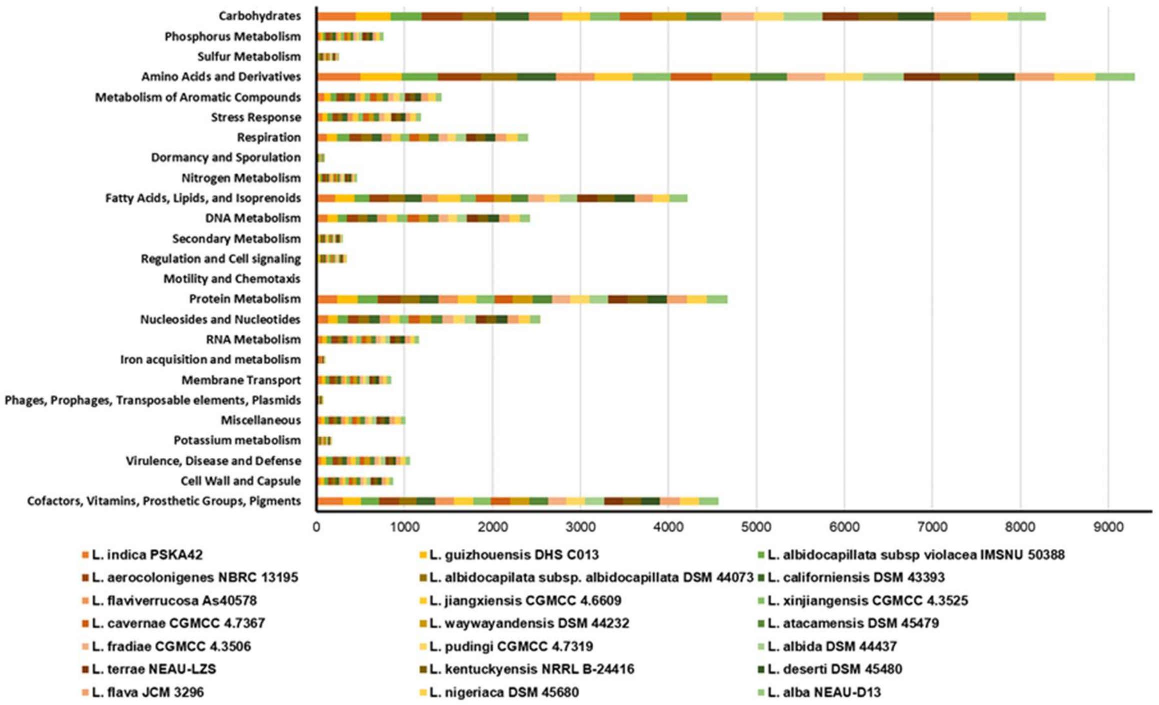

Fig. S3. Different subsystem features count according to the SEED classification of all *Lentzea* species detected by the RAST

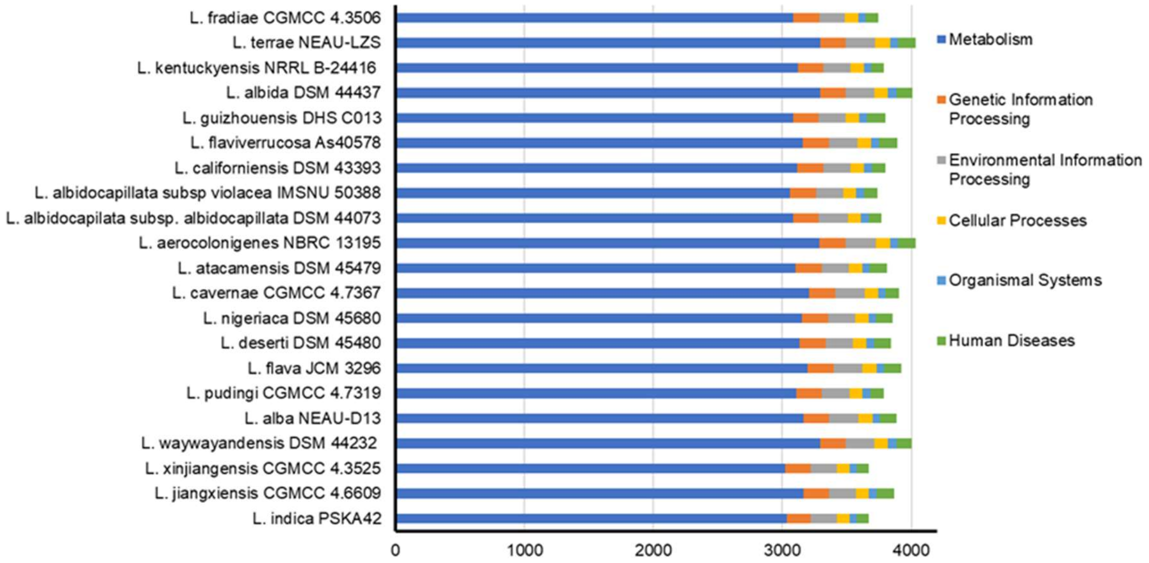

Fig. S4. Comparison of KEGG function classification amongst 21 *Lentzea* genomes

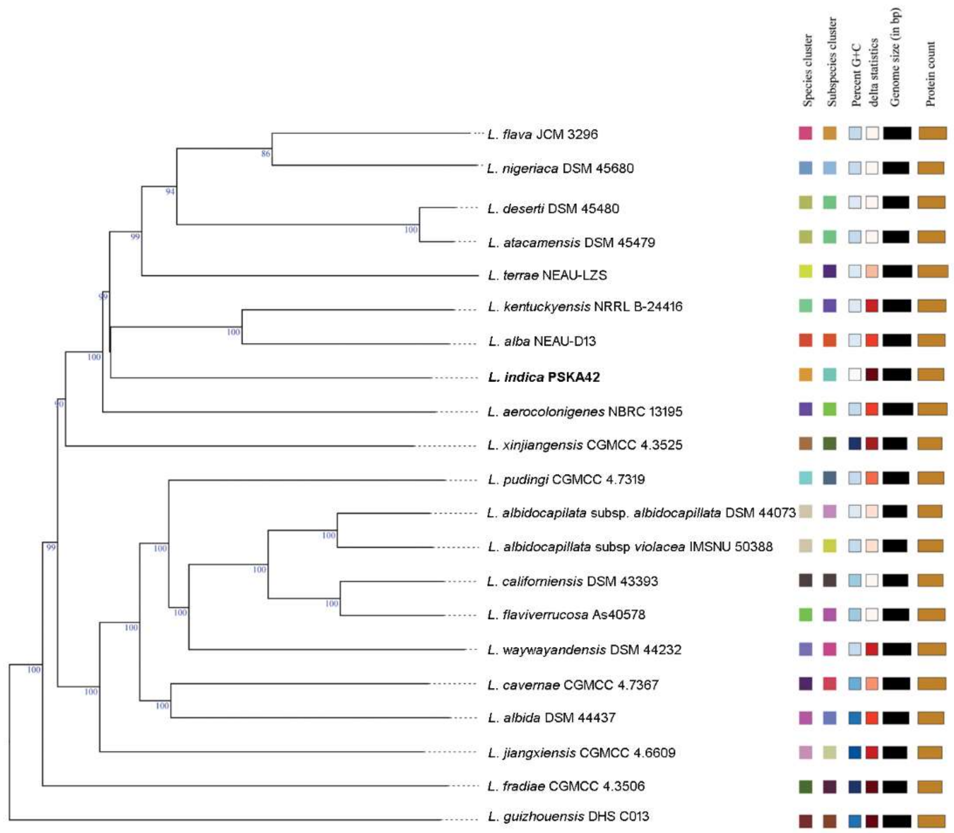

Fig. S5. Phylogenomic of strain PSKA42 and all its ancestors

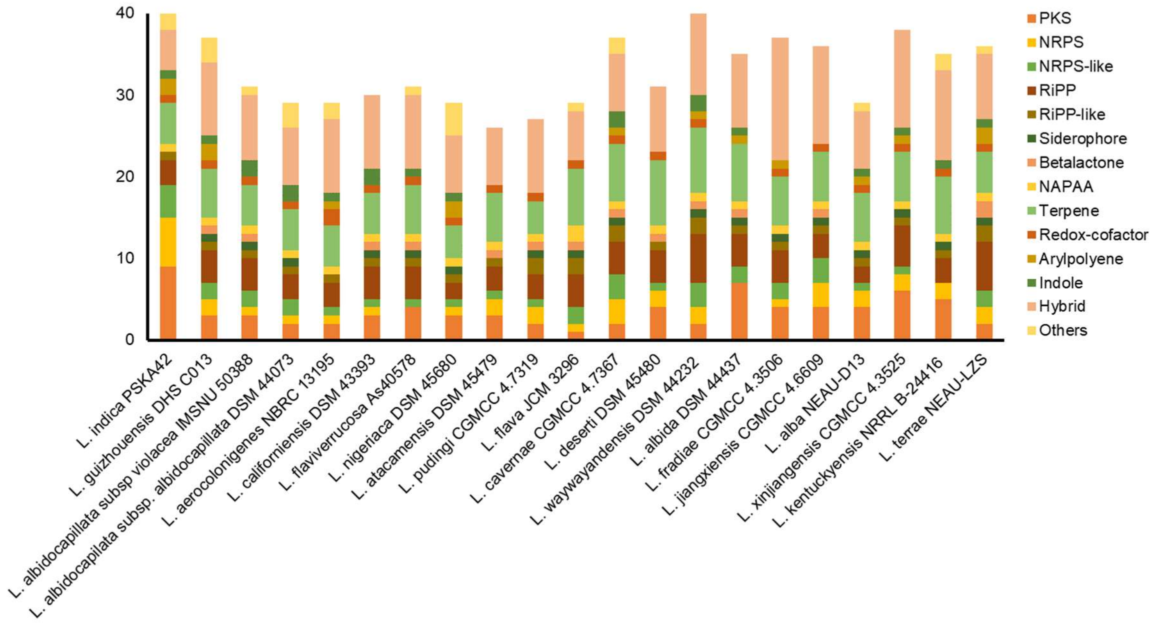

Fig. S6. BGCs distribution determined by antiSMASH in each species of *Lentzea* genome

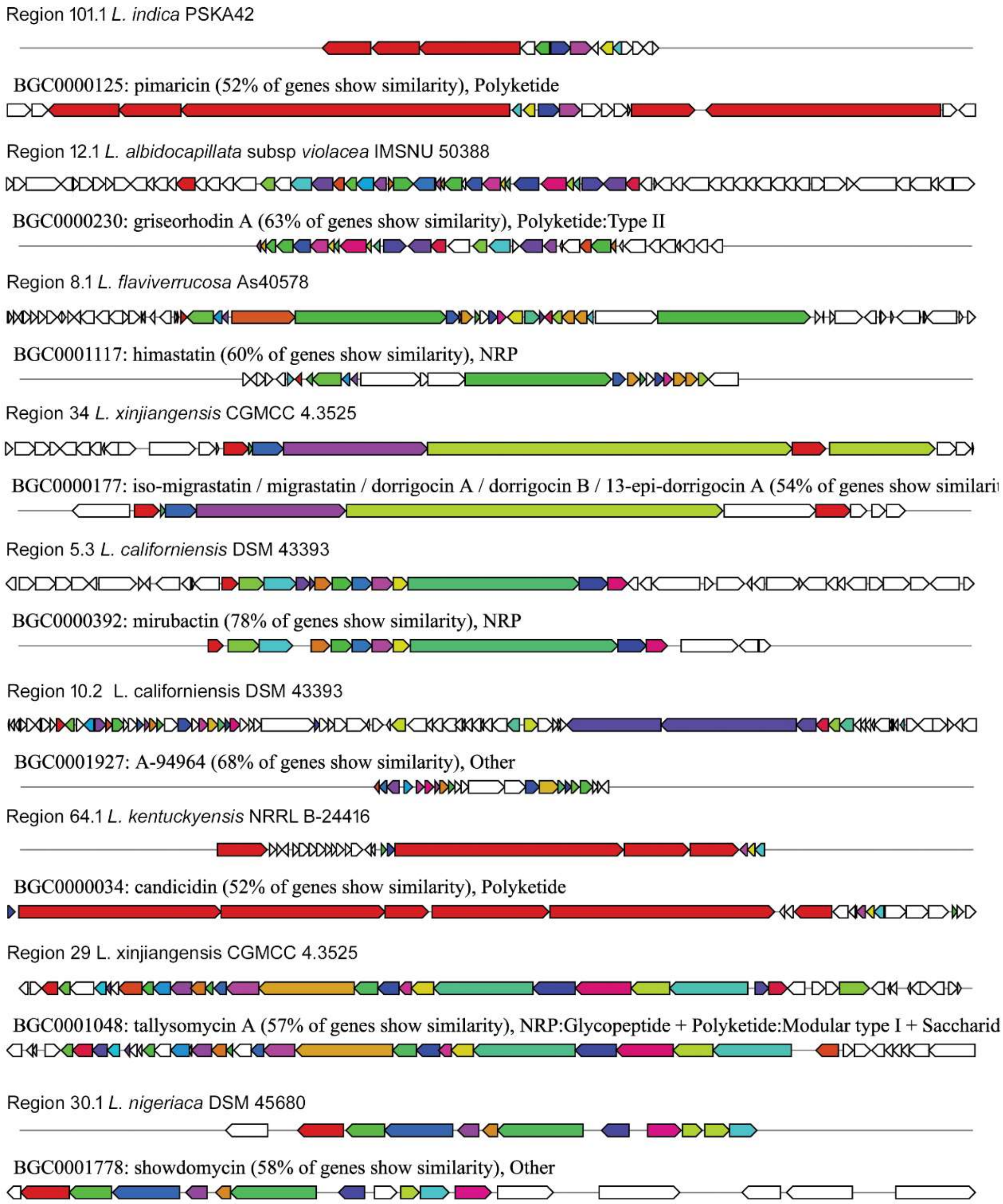

Fig. S7. Specific cluster found only in particular species

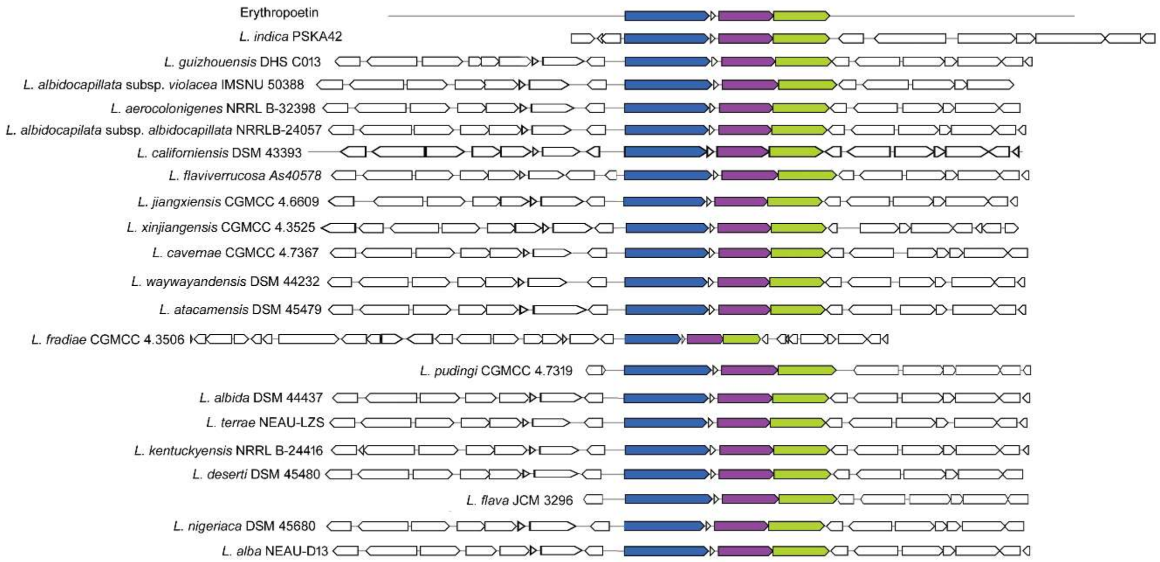

Fig. S8. Erythreapeptin-9 gene cluster from all *Lentzea* genomes

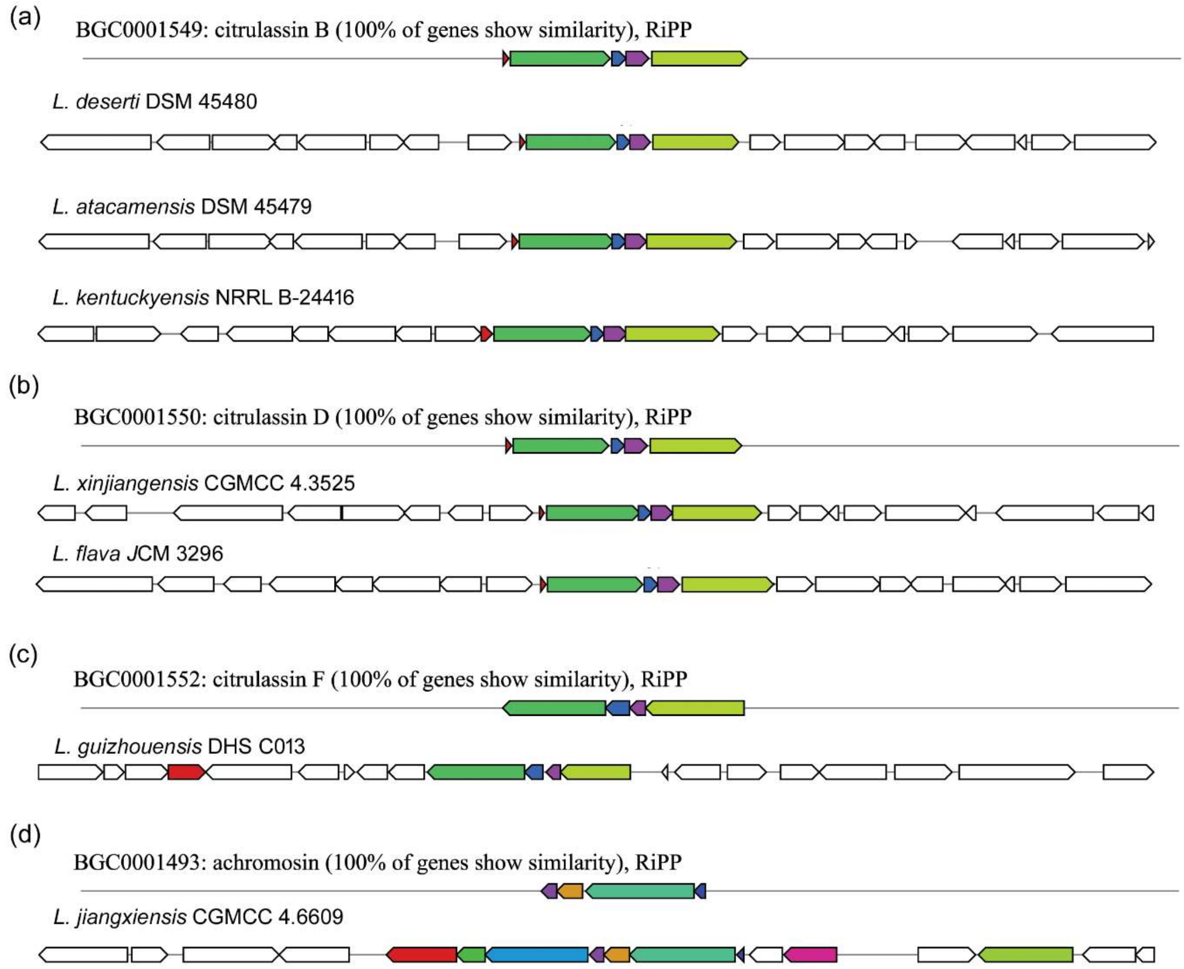

Fig. S9. Highly similar RiPPs gene cluster from *Lentzea* genomes.

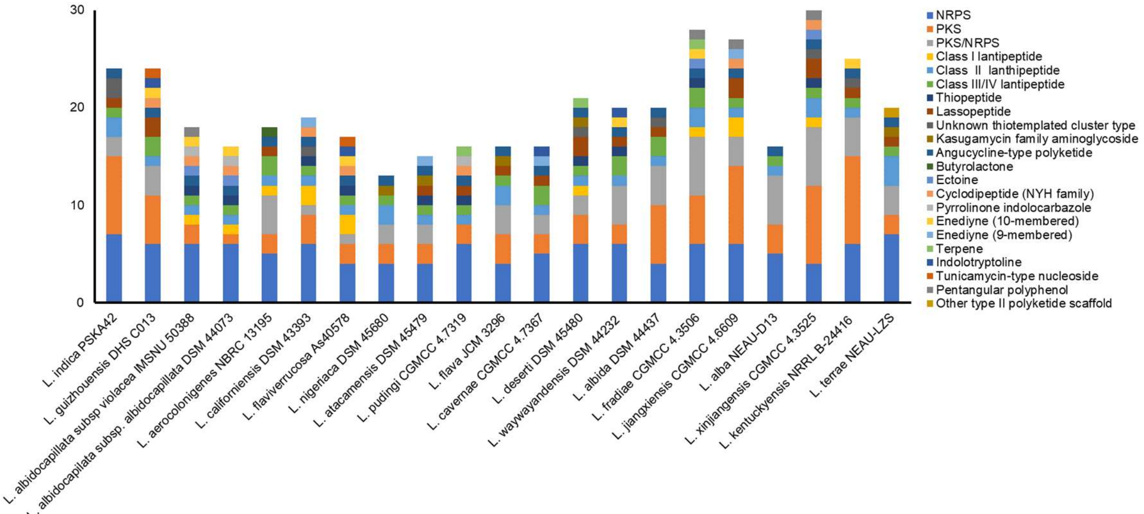

Fig. S10. Composition of BGCs detected by PRISM from *Lentzea* genomes

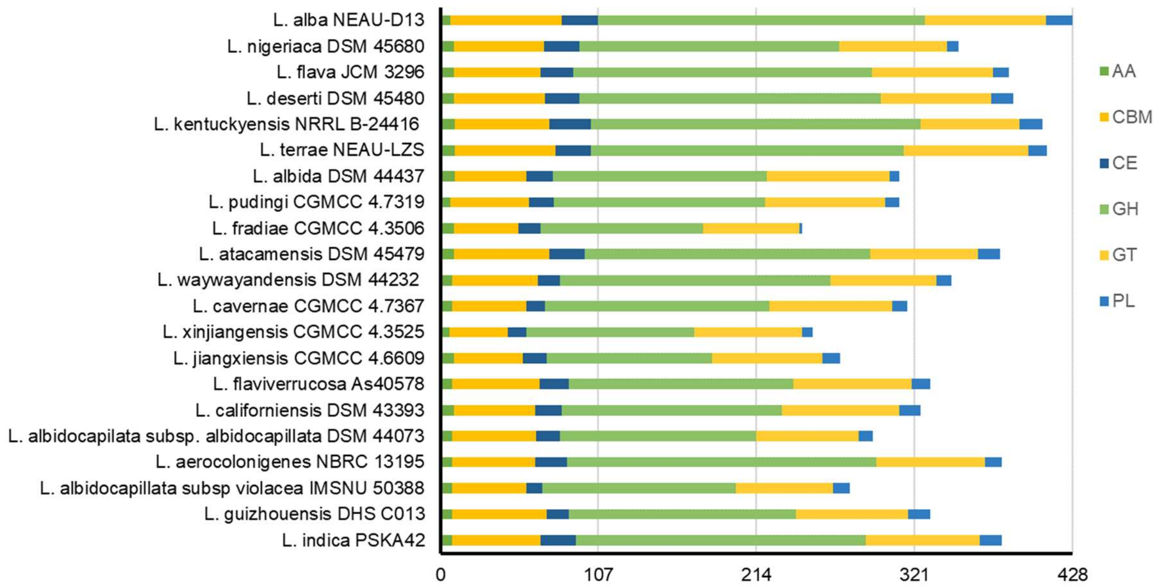

Fig. S11. Distribution of CAZymes across 21 *Lentzea* genomes

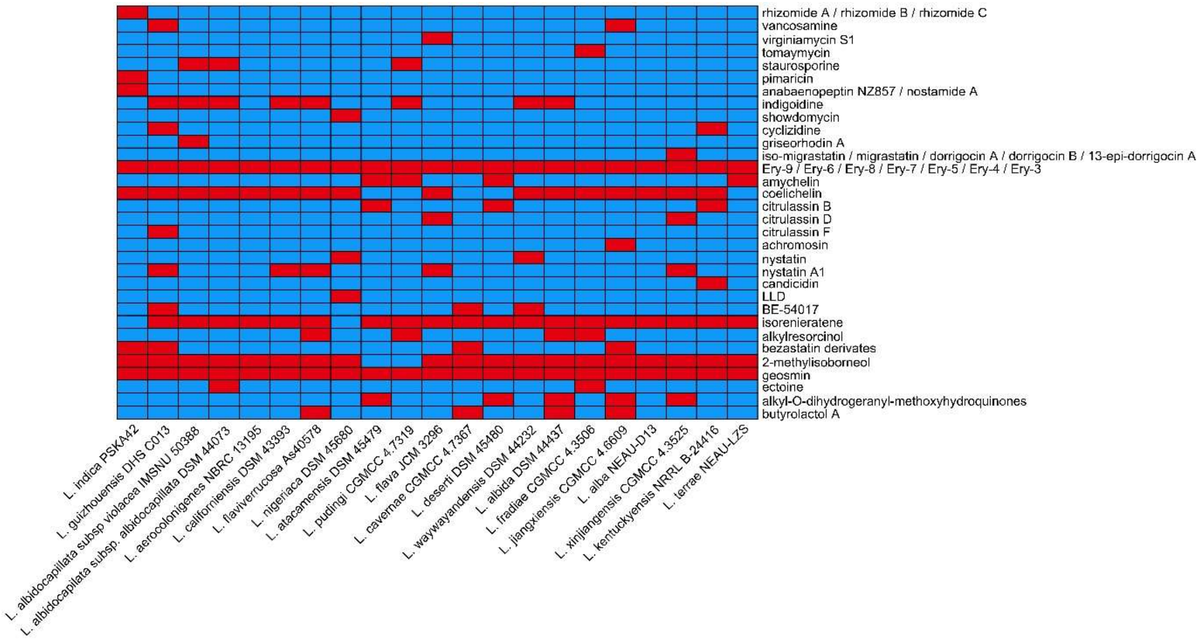

Fig. S12. Presence (red colour) and absence (blue colour) of BGCs encoded products in *Lentzea* strains as predicted by antiSMASH analysis with similarity >50-100%.
